# FcγRIIA signalling as a host-associated determinant of endosomal trafficking and antibody-dependent enhancement in flavivirus infection

**DOI:** 10.3389/fimmu.2026.1854518

**Published:** 2026-06-17

**Authors:** Nensar Wai Wai Phyo Rakwi, Daisuke Muraoka, Kazumi Haga, Adeline Syin Lian Yeo, Meng Ling Moi

**Affiliations:** 1 Department of Developmental Medical Sciences, School of International Health, Graduate School of Medicine, The University of Tokyo, Tokyo, Japan; 2 Division of Immune Response, Aichi Cancer Center Research Institute, Nagoya, Japan

**Keywords:** antibody-dependent enhancement (ADE), endosomal trafficking, FcγRIIa (CD32A), flavivirus (DENV ZIKV JEV), host-associated factors, immune complexes, pre-existing immunity, viral pathogenesis

## Abstract

**Background:**

Antibody-dependent enhancement (ADE) is a major determinant of disease severity in flavivirus infections, yet the host-associated factors that regulate the transition from antibody-mediated uptake to productive infection remain incompletely defined. Fc gamma receptor IIA (FcγRIIA/CD32A), an activating Fc receptor expressed on myeloid cells, represents a key host determinant linking pre-existing immunity to viral entry pathways. Here, we investigated how FcγRIIA signalling influences intracellular trafficking and ADE efficiency across dengue virus (DENV), Zika virus (ZIKV), and Japanese encephalitis virus (JEV).

**Method:**

Using BHK-21 cells expressing either wild-type FcγRIIA (WT) or a cytoplasmic-truncated variant lacking the immunoreceptor tyrosine-based activation motif (ITAM), we show that immune-complex internalization occurs independently of the cytoplasmic domain, but that signalling competence significantly modulates downstream infection efficiency. WT cells generally showed higher ADE efficiency, whereas cytoplasmic-truncated receptors preserved ADE activity but showed virus-dependent reductions in enhancement magnitude.

**Result:**

FcγRIIA signalling altered the intracellular routing of virus–antibody complexes. WT receptors promoted rapid trafficking from EEA1-positive early endosomes to Rab7-positive late endosomes, whereas signalling-deficient receptors displayed delayed progression and increased association with IRAP- and Rab14-positive compartments. These trafficking differences correlated with virus-specific patterns of enhancement, with DENV showing the strongest dependence on late endosomal maturation, while ZIKV and JEV utilized more flexible or alternative pathways. Transcriptomic analysis revealed minimal differences between WT and truncated receptors at later time points, indicating that FcγRIIA primarily modulates early post-entry events rather than sustained transcriptional responses.

**Conclusion:**

Collectively, these findings identify FcγRIIA signalling as a host-associated determinant that regulates the intracellular fate of virus–antibody complexes and influences ADE efficiency through modulation of endosomal trafficking dynamics. The work provides a mechanistic framework linking host receptor signalling to variability in infection outcomes under conditions of pre-existing immunity, with implications for understanding disease heterogeneity and optimizing vaccine and antibody-based interventions.

## 1 Introduction

Flaviviruses, including dengue virus (DENV), Zika virus (ZIKV), and Japanese encephalitis virus (JEV), remain major global health threats, particularly in tropical and subtropical regions (1–3). DENV alone is estimated to infect approximately 400 million individuals annually (4), while ZIKV has been linked to congenital abnormalities and neurological complications (5–7). The continued emergence and co-circulation of these viruses highlight the need to better understand the mechanisms governing infection outcomes.

A defining feature of flavivirus immunopathogenesis is antibody-dependent enhancement (ADE), in which pre-existing, non-neutralizing or sub-neutralizing antibodies facilitate increased viral entry into Fcγ receptor (FcγR)-expressing cells. This process enhances viral replication and is associated with more severe disease manifestations. Clinical and experimental evidence demonstrates that prior flavivirus exposure can exacerbate subsequent infections, as observed in DENV–ZIKV cross-reactivity and in JEV-vaccinated individuals who develop severe dengue (8–11). The most clinically significant and well-established form of ADE occurs during heterotypic secondary dengue virus infection, where pre-existing cross-reactive antibodies are associated with increased risk of severe disease manifestations (12–14).

Flavivirus entry into host cells occurs primarily via receptor-mediated endocytosis, followed by trafficking through the endosomal network (11, 15–17). Acidification within endosomes induces conformational changes in the viral envelope protein, enabling membrane fusion and release of the viral genome into the cytoplasm (18–20). The specific intracellular compartment in which fusion occurs is a critical determinant of infection efficiency, with DENV showing strong dependence on Rab7-positive late endosomes (16, 20).

ADE is mediated predominantly by FcγRs, which bind IgG-opsonized virus particles and facilitate internalization into immune cells (21, 22). Previous studies using FcγRIIA-expressing systems have shown that the FcγRIIA cytoplasmic domain and signalling competence influence ADE efficiency (23–25). FcγRs and IgG–Fc interactions regulate antibody-dependent cellular uptake, immune-complex handling, and downstream effector functions (26–29). FcγR engagement also activates downstream signalling pathways involved in cytoskeletal remodelling, phagocytosis, antibody effector function, and immune-cell activation (30–34). FcγRIIA, a major activating receptor, contains an ITAM that initiates intracellular signalling upon receptor engagement (22, 30–34). This signalling cascade regulates cytoskeletal rearrangement, endocytosis, and vesicular trafficking. While FcγRIIA-mediated uptake of virus–antibody complexes is well established, the mechanistic role of its cytoplasmic domain in regulating intracellular trafficking remains unclear. Similar FcγR-dependent effects on viral uptake and infection efficiency have also been reported in other viral systems, including Ebola virus (35). Virus–antibody complexes entering through FcγR pathways may be routed through distinct endosomal compartments, including EEA1-positive early endosomes and Rab7-positive late endosomes, which are central to viral sorting, fusion, and genome release (36–38).

In this study, we investigate the role of the FcγRIIA cytoplasmic domain in modulating intracellular trafficking during ADE. Using engineered BHK-21 cells expressing wild-type (WT) or cytoplasmic-truncated (CT) FcγRIIA, we demonstrate that while immune-complex uptake is preserved, signalling competency critically regulates endosomal progression and trafficking efficiency across DENV, ZIKV, and JEV.

## 2 Materials and methods

### 2.1 Ethical statement

This study was approved by the Institutional Review Board of the Graduate School of Medicine, the University of Tokyo (2023313NI, 2025168NI). All participants provided their written informed consent to participate in this study.

### 2.2 Cells

Baby hamster kidney cells (BHK-21, Japan Health Science Research Resource Bank), FcγRIIA-expressing BHK-21 Wild Type (WT) (25) and FcγRIIA-expressing BHK-21 without cytoplasmic region (CT) (23) were used. BHK-21 cells were cultured in Eagle’s Minimum Essential Medium (E-MEM; Sigma, St. Louis, MO, USA) supplemented with heat-inactivated 10% foetal bovine serum (FBS, Sigma) without antibiotics at 37°C in 5% CO_2_. FcγRIIA-expressing BHK-21 Wild Type (WT) and FcγRIIA-expressing BHK-21 without cytoplasmic region (CT) were cultured in E-MEM supplemented with heat-inactivated 10% FBS and 0.5mg/ml neomycin (G418; PAA Laboratories GmbH, Pasching, Austria) at 37°C in 5% CO_2_.

### 2.3 Viruses and antibodies

Dengue virus type-1 (DENV-1) 01-44-1HuNIID strain (GenBank accession no. AB111070), Zika virus (ZIKV) PRVABC59 strain (GenBank accession no. KX377337), and Japanese encephalitis virus (JEV) OH0566 strain (GenBank accession no. AY508813) were used. Viruses were propagated on BHK-21 cells at 37°C in 5% CO_2_ for 5 days. Cell culture was centrifuged at 3000 RPM for 10 min, supernatant was collected and stored in aliquots at -80°C. Virus titres (plaque-forming units (PFU) per ml) were determined by plaque assay on BHK-21 cells. Anti-flavivirus monoclonal antibody 12D11/7E and dengue-serotype-cross-reactive mouse IgG monoclonal antibody 4G2 (ATCC HB-112), which recognizes the E protein, were used in flow cytometry assays, ADE assays, and immunofluorescence assays. In addition to the two mAbs, dengue IgG-positive human serum was used for ADE assays.

### 2.4 Plasmid

**Figure 1:**
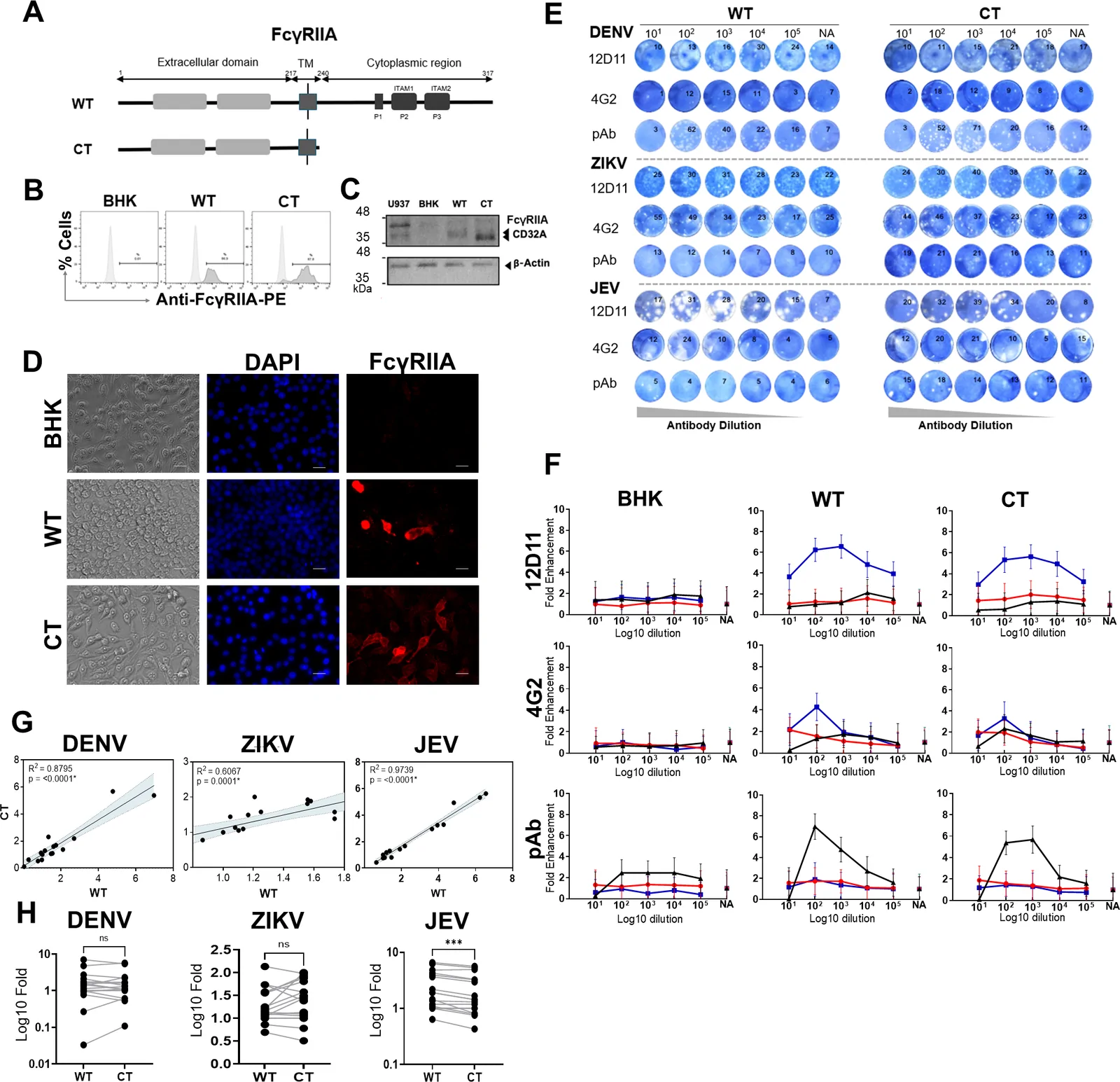
**(A)** The FcγRIIA WT open reading frame (ORF) of FcγRIIA (GenBank accession no. M31932). The CT insert was truncated at the transmembrane region and consisted of the extracellular domain and transmembrane domain. Each insert was sequenced prior to being subcloned into pcDN A3.1/neo+ (Invitrogen) and engineered constructs were generated by standard site-directed mutagenesis (QuikChange; Stratagene). Full-length sequences for the constructs were verified by DNA sequence analysis **(B)** PE-labelled monoclonal antibody to FcγRIIA was used to determine the percentage of BHK-21 cells expressing FcγRIIA. FcγRIIA-expressing BHK-21 cells, without cytoplasmic region (CT) cells that expressed at >70% were chosen for further experiments. Light grey indicates non-transfected parent BHK-21 cells. Dark grey indicates FcγRIIA expressing BHK-21 cells. **(C)** Western blot analysis of FcγRIIA. After stable transfection, the cells were harvested, lysed and the proteins were separated by a 10% SDS-PAGE. Detection was done by using anti-CD32A antibody (anti-FcγRIIA antibody). U937 cells, a myeloid cell line that expresses innate FcγRIIA (42kDa) were used as a positive control for the detection of FcγRIIA. The WT plasmid (WT) consists of the open reading frame of FcγRIIA (M31932). Molecular weight standard is as indicated. β-actin (45kDa) was used as a positive control. Non-transfected BHK cells (BHK) were used as a negative control. **(D)** To verify the transfection, the stable transfectants were immunostained with Anti-FcγRIIA-PE (Red) and visualized by Keyence BZ-X710. DAPI (Blue) is used to stain the nucleus. Scale bar 20µm. Conventional plaque assay to determine antibody-dependent enhancement (ADE) in flaviviruses (DENV, ZIKV, JEV) in FcγRIIA-expressing BHK-21 cells (WT) and FcγRIIA-expressing BHK 21 cells without cytoplasmic region (CT), using mAb 12D11 (62), mAb 4G2 (63) and dengue IgG-positive human serum (pAb). Plaque assay observations are shown in **(E)**. The fold-enhancement measured in BHK, WT, CT cells, infected with DENV, ZIKV, JEV and using mAb 12D11, mAb 4G2 and dengue IgG-positive human serum are plotted in **(F)**. The fold-enhancement values were calculated by the following formula: (mean number of plaques in the presence of antibodies or serum sample)/(mean number of plaques in the absence of antibodies or serum sample). The mean plus two standard deviations (SD) of the negative control was used as a cut off to differentiate enhancing and non-enhancing activity. DENV is indicated as black, ZIKV as red and JEV as blue. **(G)** Scatter plots display ADE activity between WT and CT cells across matched antibody or serum conditions. Positive correlations indicate that WT and CT cells preserved similar ADE response patterns across matched conditions, while direct paired comparisons were used to assess differences in enhancement magnitude. **(H)** Paired comparison of ADE activity between WT and CT cells across matched antibody or serum conditions. Each connected pair represents the same antibody dilution or serum condition tested in WT and CT cells, and ADE values are shown on a log10 scale. Statistical analysis was performed using a two-tailed paired t-test.

Human FcγRIIA cDNA was provided by Dr. Jeffrey V. Ravetch, Rockefeller University, NY, USA. The WT insert codes for the open reading frame (ORF) of FcγRIIA (GenBank accession no. M31932, protein_id=AAA35827.1). The CT insert was truncated at the transmembrane region and consisted of the extracellular domain and transmembrane domain (**Figure 1A**). Each insert was sequenced before being subcloned into pcDNA3.1/neo+ (Invitrogen) and engineered constructs were generated by standard site-directed mutagenesis (QuikChange; Stratagene). Full-length sequences for the constructs were verified by DNA sequence analysis (23). Primers used for insert verification are listed in **Supplementary Table 1**.

### 2.5 Transfection of FcγRIIA constructs

Transfection of BHK-21 cells with WT or engineered FcγRIIA cDNA was carried out with Lipofectamine LTX (Invitrogen), according to the manufacturer’s protocol. Cells were seeded one day before transfection and on the next day, at 50~70% confluency, the DNA-lipid complex was prepared and incubated for 30 minutes at room temperature. After lipofectamine transfection, the cells were incubated at 37°C 5% CO_2_ for up to 24 hours. The cells were then examined for surface expression of FcγRIIA by flow cytometry, using PE-conjugated Fc gamma RII/CD32 (#FAB1330P, R&D, Minneapolis, USA) and standard immunoblot analysis at 48 hours following transfection.

The cells were passaged in neomycin and then selected for stable plasmid expression. Selected cells were passaged in neomycin (0.8 mg/ml) for up to 8 passages. BHK-21 transfectants were washed with PBS (Invitrogen) and stained with phycoerythrin (PE)-conjugated mAb to human FcγRIIA (CD32A mAb, clone 190723; R&D Systems), according to the manufacturer’s instructions. Stained cells were analysed using a Guava EasyCyte Mini cytometer (Millipore, Billerica, MA, USA). More than 5000 events were obtained for each sample, and the results were analysed by using FlowJo Version 10.9.0 software (BD Life Sciences, Franklin Lakes, NJ, USA). Transient expression of WT and CT in BHK-21 cells was examined by flow cytometry. For determining DENV, ZIKV, and JEV infection by immunofluorescence method (IFA), the cells were fixed and permeabilized with 1: 1 acetone: methanol mixture for 10 min and stained with mAb 4G2 at 37°C for 60 min. The cells were then washed in PBS and stained with a secondary anti-mouse antibody (Alexa Fluor™ 488 Antibody, Invitrogen). The cells were then observed under fluorescence microscopy (Keyence BZ-X710, Keyence Corp., Osaka, Japan).

### 2.6 Infection assay

Monoclonal antibodies 12D11 and 4G2 were serially diluted 10-fold from 1:10^1^ to 1:10^5^ with E-MEM supplemented with 2% FBS for each experiment. The serum sample was also serially diluted in the same manner. The virus–antibody complex was prepared by mixing 50µl of DENV, ZIKV, and JEV at titres of 20~50 PFU with 50µl of diluted serum samples, diluted mAb 12D11, mouse mAb 4G2 supplemented with serum-free E-MEM. Virus–antibody mixture was incubated at 37°C for 60 min before using in infection experiments.

After stable cell-line establishment, ADE activity was determined using a conventional plaque assay (25). Infection assay was done at 70~80% confluency of BHK-21 cells (control), WT and CT monolayer 12 well plates, respectively. Using serial dilutions of antibodies with DENV, ZIKV, and JEV. The viral immune complexes were prepared as mentioned above and the cells were infected 100 µl per well. After 1 hour of infection, cells were overlayed with a low IgG Methylcellulose overlay medium. After 4–5 days post-infection, the cells were fixed and stained to count the plaques.

### 2.7 Immunofluorescence assay and colocalization with endosomal markers

Cells were seeded in 10% FBS E-MEM on glass slides at 2 x 10^5^ cells on 8 well glass slides the day prior to experiment. Cells were then briefly washed with 1x D-PBS and starved with serum depleted E-MEM for 10–12 hours. The virus immune complex is prepared, using DENV, ZIKV, JEV at MOI 0.1 and dengue IgG-positive human serum or mAb 4G2 at 1:100 dilution. The complex is incubated at 37°C shaking 150 rpm for 1 h. Next, cells were cooled to 4°C for 10 min prior to infection assay. After infection, the cells were incubated at 37°C. At exact time points, 5 min and 30min, the cells were shifted to 4°C on ice and proceeded to fix, permeabilize and immunostained with primary antibodies; Anti-FCGR2A/Clone OTI9C6 (#TA500645S, Origene), anti-EEA-1 (#2411S, Cell Signalling Technology), anti-IRAP (#68187, Cell Signalling Technology), anti-Rab14 (#R0656, Merk), anti-Rab7 (D95F2 XP) (#93671T, Cell Signalling Technology). The endosomal markers used in this study and their functional relevance are summarized in **Supplementary Table 2**. For secondary antibodies, Alexa fluor 488 goat anti-mouse antibody (#A11001, Invitrogen) and R-PE goat anti-rabbit antibody (#SA00008-2, Proteintech) were used. DAPI (#19178-91, e-Nacalai) was used to stain the nucleus. Stained slides were mounted with anti-fade (in-house) and stored at 4°C. The images are visualized by Keyence BZ-X710 with 60x oil immersion lens. Colocalization analysis was performed using the original-resolution Keyence image files in BZ-X Analyzer and Hybrid Cell Count software. Background subtraction and fluorescence thresholding were applied using consistent parameters across samples within each virus and marker set. The reduced-size representative images shown in the figures were used for visualization, whereas quantitative analyses were performed on the original image files. The percentage of colocalization of CD32A with endosomal markers was determined using the same intensity thresholds for green and red signal extraction across all samples to minimize potential bias. FcγRIIA-non-expressing BHK-21 cells were included as visual negative controls and excluded from the statistical interpretation of FcγRIIA-based colocalization.

### 2.8 RNA sequencing analysis

Cells were seeded in 10cm dishes and infected with DENV, ZIKV, JEV under ADE conditions using a dengue IgG-positive human serum. The total RNA from 63 samples was extracted at 24- and 48-hour post infection (hpi) by using the RNeasy Kit (Qiagen), and the RNA yield and quality was assessed using Nanodrop spectrophotometer. Each sample set consist of 3 replicates. Library construction was performed by using xGen RNA Library Prep kit (#1009814, IDT) with xGen CDI Primers (#109918615, IDT), according to manufacturer’s instructions. The libraries were pooled in equimolar concentrations based on quantification by Qubit fluorometer. Fragments of appropriate size (approximately 300 bp including adapters) were selected using AMPure XP beads to ensure consistency across the libraries. Pooled library size was confirmed by gel electrophoresis. Pooled libraries were subjected to paired end 150 bp sequencing on the Illumina NovaSeq 6000 platform. Approximately 20–30 million reads per sample were generated for each sample. Raw reads were trimmed with BBDuk and mapped to the reference sequence of Mesocricetus auratus (golden hamster) genome (GCF_017639785.1). Expression levels of annotated sequences were quantified, and differential gene expression analysis was performed in R (v4.2.2) using the DESeq2 package (v1.38.3) with three biological replicates per group. Volcano plots were generated using the EnhancedVolcano package (v1.16.0) to visualize significantly up- and down-regulated genes, and heatmaps of the top 500 most variable genes were created using pheatmap (v1.0.12) after variance-stabilizing transformation of filtered count data. Gene Ontology (GO) enrichment analysis for biological processes was conducted using clusterProfiler (v4.6.0), with gene symbols converted to Entrez IDs via org.Mm.eg.db (v3.15.0). Enriched GO terms were visualized using dot plots.

### 2.9 Data analysis

Simple linear regression was performed using GraphPad Prism version 10.2.0, GraphPad Software, Boston, Massachusetts USA. Correlations of enhancement activity between WT and CT, across DENV, ZIKV, and JEV using mAb 12D11, 4G2, and dengue Ig G positive human serum, are plotted with a coefficient of determination (r), with a p-value <0.05 as significant (*) and 95% confidence interval. Statistical comparison between WT and CT groups was performed using Mann–Whitney test as appropriate. Paired comparison of matched WT and CT ADE values in **Figure 1H** was performed using a two-tailed paired t-test. Western blot images are developed using Luminograph I and quantification by ImageJ Software. Quantification of the colocalization analysis was done using BZ-Analyzer (Hybrid cell count, Macro cell count) Keyence BZ X-710. Two-way ANOVA analysis was performed by using GraphPad Prism version 10.5.0, GraphPad Software, Boston, Massachusetts USA.

## 3 Results

### 3.1 Stable expression of transfected cells

Expression levels of WT and CT in BHK-21 cells were determined by flow cytometry. PE-labelled monoclonal antibody to FcγRIIA was used to determine the percentage of BHK-21 cells expressing FcγRIIA. After 8 passages, the percentage of cells expressing FcγR WT and CT was 86.9% and 87.8%, respectively. Cell lines that had over 70% were then chosen for further experiments. The expression of the WT and CT constructs in BHK-21 cells was verified by immunoblotting and immunofluorescence staining (**Figures 1A–D**).

### 3.2 ADE activity across flaviviruses

Conventional plaque assay was used to determine antibody-dependent enhancement (ADE) in flaviviruses (DENV, ZIKV, JEV) in FcγRIIA expressing BHK-21 cells (WT) and FcγRIIA expressing BHK-21 cells without cytoplasmic region (CT), using mAb 12D11, mAb 4G2 and dengue IgG-positive human serum (**Figure 1E**). Fold enhancement values were calculated using the following ratio: (mean number of plaques in the presence of antibodies or serum samples)/(mean number of plaques in the absence of human serum samples, negative control). The sum of the mean of the negative control plus two times the standard deviation (SD) value obtained from 4 wells of negative control was used as a cut-off to differentiate enhancing and non-enhancing activity (14, 39, 40) (**Figure 1F**). Initial results have demonstrated differential ADE patterns across flaviviruses by using mAb 12D11, mAb 4G2 and dengue IgG-positive human serum. Initial ADE assays demonstrated distinct virus- and antibody-dependent enhancement patterns across DENV, ZIKV, and JEV using mAb 12D11, mAb 4G2, and dengue IgG-positive human serum. With mAb 12D11, JEV showed the highest enhancement among the three flaviviruses, with peak FE values of 4.89 in WT cells and 5.47 in CT cells. With mAb 4G2, JEV also showed higher enhancement than DENV and ZIKV, with peak FE values of 4.27 in WT cells and 3.29 in CT cells. In contrast, dengue IgG-positive human serum produced the strongest enhancement with DENV, with peak FE values of 10.0 in WT cells and 6.40 in CT cells. These results indicate that ADE magnitude differed according to both virus type and antibody source. Absolute plaque counts underlying the DENV ADE fold-enhancement values for mAb 12D11/7E, mAb 4G2, and dengue IgG-positive human serum are provided in **Supplementary Tables 3**–**5**.

Correlations of enhancement activity between WT and CT, across DENV, ZIKV, JEV using mAb 12D11, 4G2, and dengue IgG-positive human serum, are plotted with a coefficient of determination (R^2^), with a p-value <0.05 as significant (*) and 95% confidence interval (**Figure 1G**). Linear regression showed significant correlations of WT and CT across DENV, ZIKV, JEV, with R^2^ = 0.8795, p = <0.0001; R^2^ = 0.6067, p = 0.0001; R^2^ = 0.9739, p = <0.0001, respectively. ADE was observed in both WT and CT cells, with a consistent trend toward higher enhancement efficiency in WT cells, indicating that FcγRIIA signalling modulates the magnitude rather than the occurrence of enhancement. Direct paired comparison of matched WT and CT values showed no significant difference in overall ADE magnitude for DENV and ZIKV, whereas JEV showed significantly higher enhancement in WT cells, indicating a virus-dependent contribution of the FcγRIIA cytoplasmic region to ADE efficiency (**Figure 1H**).

### 3.3 Intracellular trafficking of immune complexes via EEA-1-FcγRIIA mediated virus immune complex intake

**Figure 2:**
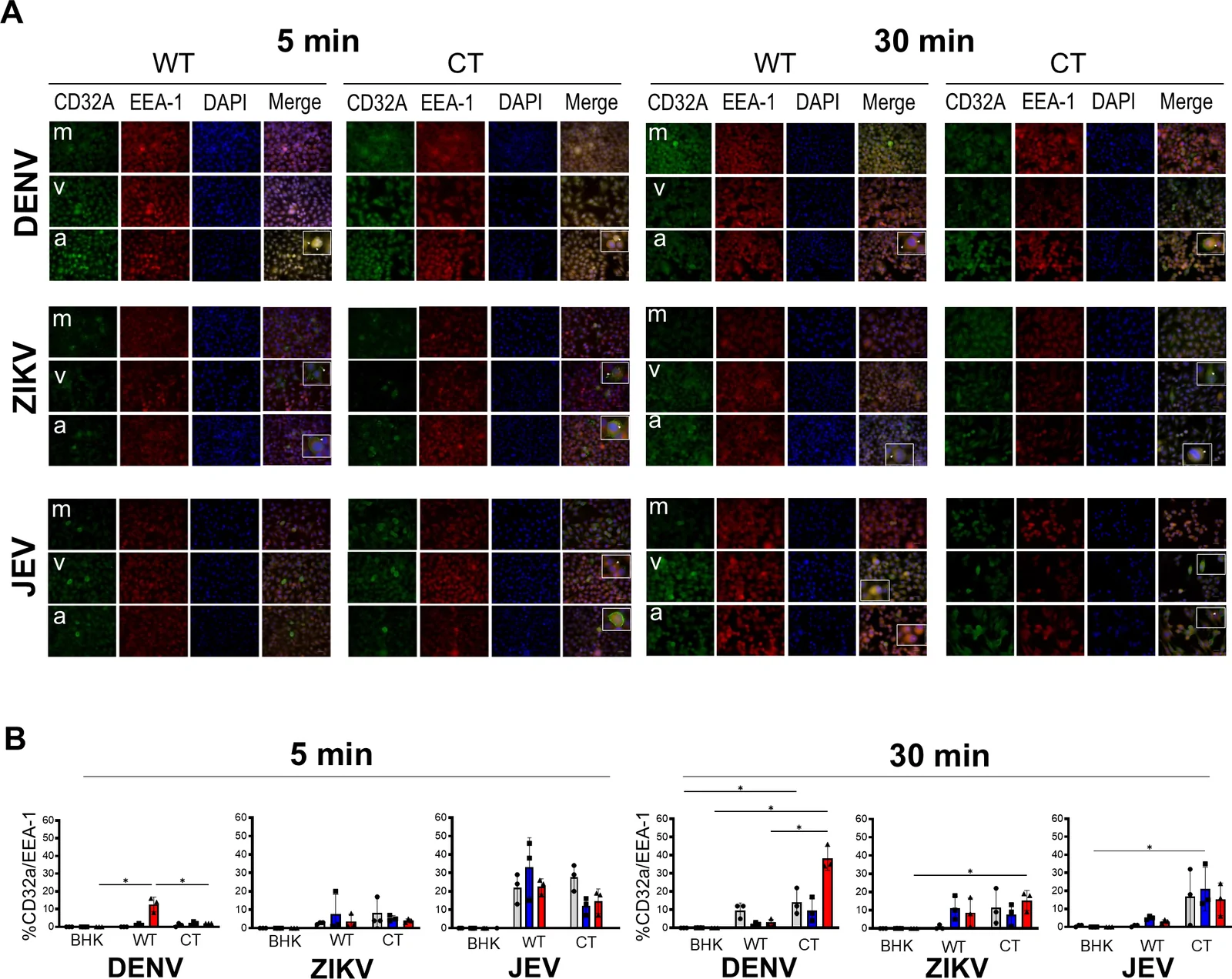
Colocalization of CD32A and EEA-1 in BHK, WT, and CT cells under DENV, ZIKV, JEV infection and ADE conditions using dengue IgG-positive human serum, at 5 min- and 30 min- post-infection. Representative immunofluorescence images of wild-type FcγRIIA-expressing BHK-21 cells (WT), FcγRIIA-expressing BHK-21 cells lacking the cytoplasmic region (CT) at 5 min post-infection and 30 min post-infection under DENV, ZIKV, JEV ADE conditions **(A)**. Cells were treated under mock (m), virus-only (MOI 0.1) (v), or ADE conditions (DENV/ZIKV/JEV, MOI 0.1 with dengue IgG-positive human serum at 1:100 dilution) (a). Colocalization was analysed using Keyence BZ-X Hybrid Cell Count Software by calculating the percentage of CD32A-positive signal overlapping with the EEA-1 signal **(B)**. Quantitative colocalization analysis was performed using the original-resolution image files; representative images were resized for figure presentation. Thresholds for each fluorescence channel were set consistently across all groups within each time point to minimize potential bias. Data represent analysis of three different fields per condition from one experiment. Statistical analysis was performed in GraphPad Prism using two-way ANOVA with *post-hoc* tests, and significance is indicated by asterisks (*p < 0.05). (See **Supplementary Figure 1** for corresponding BHK images.).

To investigate the early intracellular trafficking of virus-immune complexes during ADE conditions, we examined colocalization between CD32A (FcγRIIA) and EEA-1 (an early endosome marker) at 5- and 30-min post-infection (**Figure 2**). The immunofluorescence assay was performed in FcγRIIA-expressing BHK-21 cells (WT), FcγRIIA-expressing BHK-21 cells, without the cytoplasmic region (CT), and FcγRIIA non-expressing BHK-21 cells (BHK) infected with DENV, ZIKV, or JEV under ADE conditions using dengue IgG-positive human serum. Colocalization was quantified as the percentage of CD32A signal overlapping with EEA1.

Under ADE conditions with DENV, WT exhibited statistically strong colocalization between CD32A and EEA-1 at 5 min post-infection, indicating rapid internalization of virus–antibody complexes into early endosomes, whereas CT showed significantly lower overlap signal at this early timepoint, and BHK showed minimal colocalization. By 30 min post-infection, WT showed a decrease in colocalization, consistent with possible progression from early to late endosomes. Conversely, CT showed a significant increase in colocalization at 30 min, suggesting delayed trafficking in the absence of the cytoplasmic domain. Quantification of CD32A–EEA1 colocalization is shown in the bar graph. Representative BHK-cell images for each endosomal marker are shown in **Supplementary Figure 1**. These findings suggest that the cytoplasmic domain of FcγRIIA promotes efficient early endosomal entry of DENV immune complexes.

In ZIKV ADE conditions, colocalization at 5 min was minimal across all groups, WT, CT, BHK, respectively. By 30 min, CT showed higher EEA-1 overlap. However, this level did not exceed the CT mock baseline significantly, indicating no infection-related increase in early endosomal targeting under ZIKV ADE infection. WT remained low at 5 min and showed only a slight change by 30 min. The observed shift suggests that ZIKV immune complexes seem to be internalized rather slowly and may reach early endosomes through a possible partial FcγRIIA-independent route. The colocalization percentage is summarized in bar graphs.

Similar to DENV, JEV under ADE condition observed higher colocalization of CD32-EEA-1 in WT and CT at 5 min post-infection. This was followed by a decreasing trend in colocalization at 30 min post-infection for WT, and CT remained unchanged. On the other hand, infection with JEV alone showed comparatively higher colocalization of CD32A-EEA-1 than those under ADE conditions. Importantly, the overlap percentage of ADE condition in CT was not higher than that of CT Mock, consistent with limited ADE-specific engagement of early endosomes in this setting. This pattern was consistent across both WT and CT cells but was not statistically significant. Across all three flaviviruses, BHK cells demonstrated minimal CD32A–EEA1 colocalization, confirming that the observed trafficking is dependent on FcγRIIA expression. Collectively, these observations highlight that the cytoplasmic region of FcγRIIA plays a key role in determining the speed and efficiency of early endosomal targeting during ADE. While DENV trafficking relies on fast, signalling-dependent endosomal delivery, ZIKV and JEV may utilize alternative or delayed internalization routes that remain partially functional even in the absence of FcγRIIA signalling. To verify the EEA-1 colocalization observations with CD32A using dengue IgG-positive human serum, the colocalization experiment was confirmed by using the pan-flavivirus monoclonal antibody, mAb 4G2 with ZIKV (**Supplementary Figure 2**). The BHK, WT and CT cells were infected under ADE conditions, using ZIKV and mAb 4G2. Cells were fixed at 5 min- and 30 min- post-infection timepoints. Consistently, CT cells showed increased colocalization of CD32A in the EEA-1 compartment at 30 mins post-infection under ADE conditions. This finding supports the idea that, in the absence of FcγRIIA signalling, the opsonized immune complexes tend to persist longer in early endosomes.

### 3.4 CD32A–IRAP colocalization suggests limited involvement in ADE trafficking

**Figure 3:**
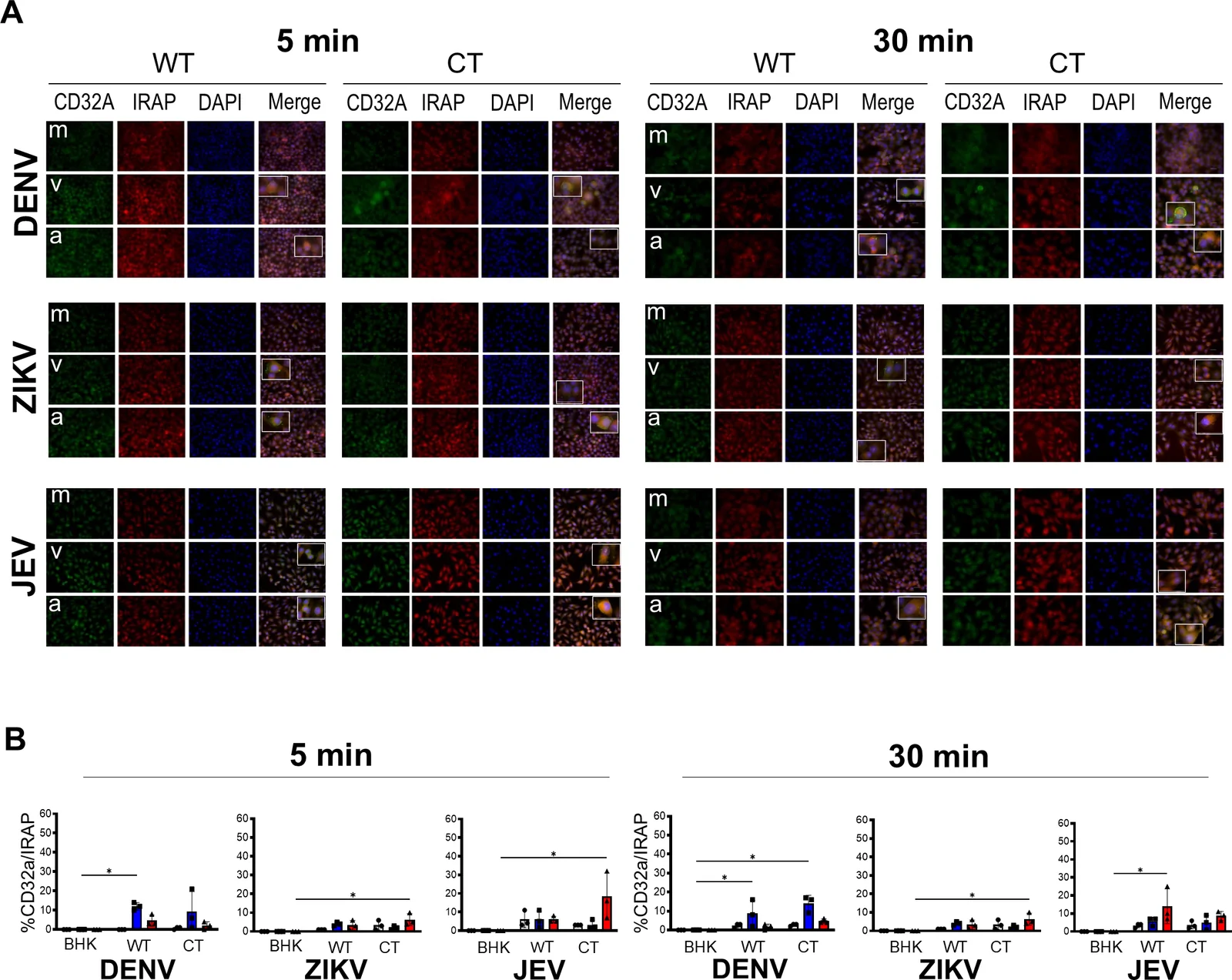
Colocalization of CD32A and IRAP in BHK, WT, and CT cells under DENV, ZIKV, JEV infection and ADE conditions using dengue IgG-positive human serum, at 5 min- and 30 min- post-infection. Representative immunofluorescence images of full length FcγRIIA-expressing BHK-21 cells (WT), FcγRIIA-expressing BHK-21 cells lacking the cytoplasmic region (CT) at 5 min post-infection and 30 min post-infection under DENV, ZIKV, JEV ADE conditions **(A)**. Cells were treated under mock (m), virus-only (MOI 0.1) (v), or ADE conditions (DENV/ZIKV/JEV, MOI 0.1 with dengue IgG-positive human serum at 1:100 dilution) (a). Colocalization was analysed using Keyence BZ-X Hybrid Cell Count Software by calculating the percentage of CD32A-positive signal overlapping with the IRAP signal **(B)**. Quantitative colocalization analysis was performed using the original-resolution image files; representative images were resized for figure presentation. Thresholds for each fluorescence channel were set consistently across all groups within each time point to minimize potential bias. Data represent analysis of three different fields per condition from one experiment. Statistical analysis was performed in GraphPad Prism using two-way ANOVA with *post-hoc* tests, and significance is indicated by asterisks (*p < 0.05). (See **Supplementary Figure 1** for corresponding BHK images.).

To explore whether the virus-immune complexes are routed through non-canonical pathways when FcγRIIA signalling is impaired, we examined colocalization between CD32A and IRAP, a marker of specialized endosomes involved in recycling and immune signalling at 5min- and 30 min- post-infection (**Figure 3**). The immunofluorescence assay was performed in FcγRIIA-expressing BHK-21 cells (WT), FcγRIIA-expressing BHK-21 cells, without cytoplasmic region (CT), and FcγRIIA non-expressing BHK-21 cells (BHK) infected with DENV, ZIKV, or JEV under ADE conditions using dengue IgG-positive human serum. Colocalization was quantified as the percentage of CD32A signal overlapping with IRAP.

In DENV infection, IRAP colocalization was low across most condition timepoints in both WT and CT. Of note, WT and CT cells under non-enhancing conditions showed significantly elevated IRAP colocalization with CD32A up to 30 min post-infection. However, no significant increase in signal overlap was observed in either WT or CT cells under ADE conditions. This suggests that DENV particles may transiently pass through the IRAP-positive compartments in the absence of antibody opsonization. In contrast, ADE conditions did not show such overlapping signals, indicating that antibody-mediated entry may bypass IRAP-associated routes. These findings imply that while IRAP is unlikely to be a major contributor to FcγRIIA-mediated ADE trafficking in DENV infection, it may serve a minor role during non-ADE virus uptake.

During ZIKV infection, WT had a significant increase in CD32A–IRAP colocalization under ADE conditions at 30 min. While overall IRAP colocalization remained limited in CT cells during ZIKV infection, transient increases observed under selected antibody conditions suggest that alternative trafficking pathways may still occur in the absence of FcγRIIA cytoplasmic signalling. This suggests that, in the presence of the FcγRIIA cytoplasmic region, ZIKV–antibody complexes may be routed to IRAP-positive compartments during later stages of internalization. The absence of similar findings in CT cells supports the hypothesis that the trafficking may require an intact cytoplasmic region. Although the functional outcome of IRAP involvement in ZIKV infection remains unclear, this delayed but notable colocalization in WT raises the possibility that IRAP-associated endosomes contribute to virus processing in a signalling-dependent manner.

A similar trend was observed in JEV infection, where IRAP colocalization remained low in WT and BHK cells at both timepoints. However, CT had elevated IRAP colocalization at 5 min under ADE conditions, suggesting that in the absence of FcγRIIA signalling, JEV–antibody complexes may transiently access IRAP-positive compartments early during infection. While the biological relevance of this early engagement is not yet clear, it raises the possibility that alternative, signalling-independent trafficking routes might be active in CT cells, particularly during JEV ADE. Overall, these findings indicate that IRAP-positive compartments may be engaged during ZIKV and JEV ADE in a virus- and FcγRIIA-signalling-dependent manner, but not for DENV, which appears to bypass this compartment entirely. Taken together, these findings indicated that IRAP involvement in ADE trafficking is virus-specific and dependent on FcγRIIA signalling, with ZIKV showing potential engagement of IRAP-positive compartments in WT, while JEV exhibits transient IRAP access in CT. To confirm the CD32A and IRAP overlap findings with dengue IgG-positive human serum, we detected the colocalization of CD32A with IRAP using the pan-flavivirus monoclonal antibody, mAb 4G2, with ZIKV under ADE conditions (**Supplementary Figure 2**). Cells were fixed in the same manner as before. IRAP colocalization was higher in CT cells at 5 min, consistent with the human serum-based observations and suggesting early engagement of IRAP-positive compartments in the absence of the FcγR cytoplasmic region.

### 3.5 CD32A–Rab14 colocalization indicates virus- and cell type-specific engagement of alternative trafficking routes

**Figure 4:**
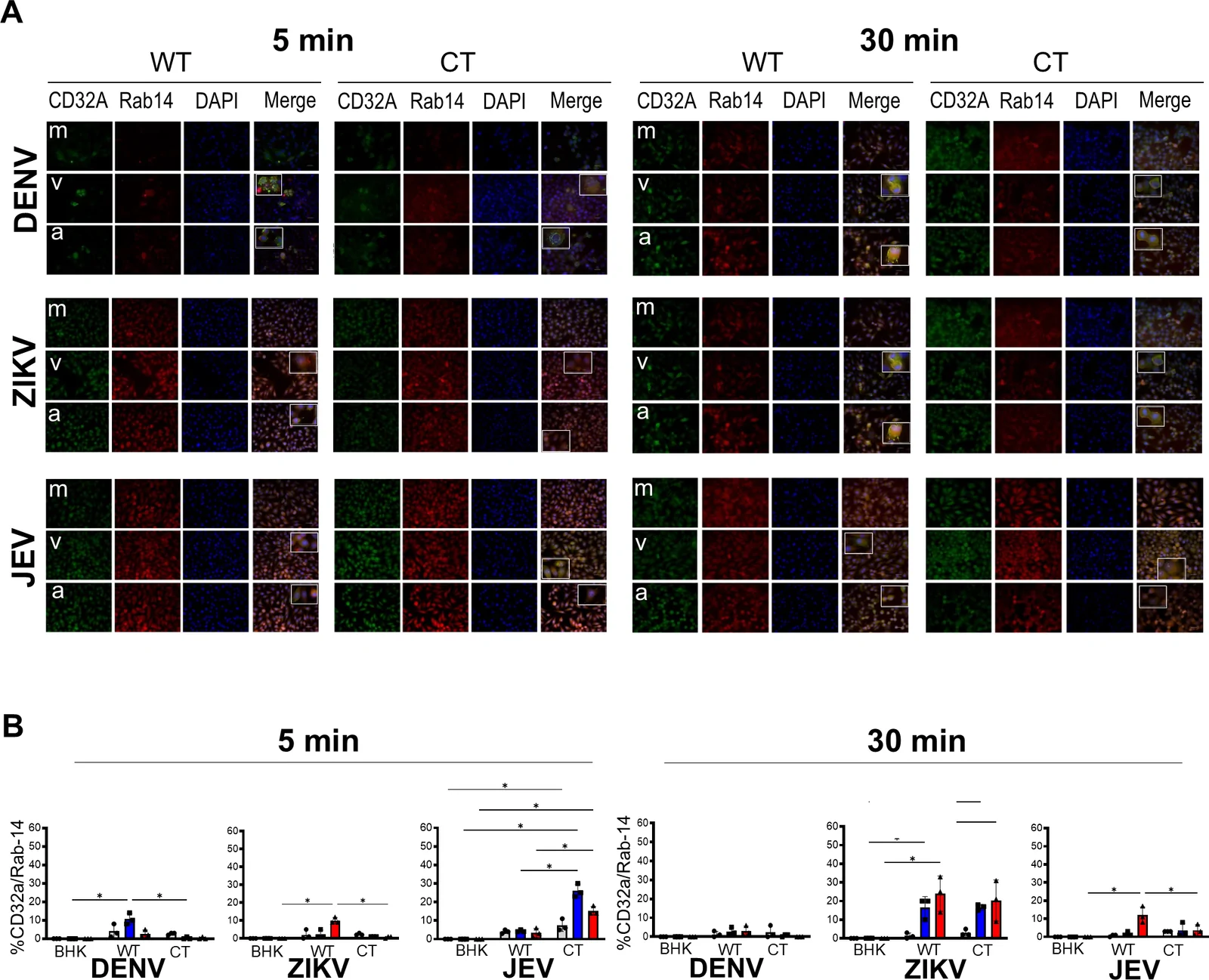
Colocalization of CD32A and Rab14 in BHK, WT, and CT cells under DENV, ZIKV, JEV infection and ADE conditions using dengue IgG-positive human serum, at 5 min- and 30 min- post-infection. Representative immunofluorescence images of full length FcγRIIA-expressing BHK-21 cells (WT), FcγRIIA-expressing BHK-21 cells lacking the cytoplasmic region (CT) at 5 min post-infection and 30 min post-infection under DENV, ZIKV, JEV ADE conditions **(A)**. Cells were treated under mock (m), virus-only (MOI 0.1) (v), or ADE conditions (DENV/ZIKV/JEV, MOI 0.1 with dengue IgG-positive human serum at 1:100 dilution) (a). Colocalization was analysed using Keyence BZ-X Hybrid Cell Count Software by calculating the percentage of CD32A-positive signal overlapping with the Rab14 signal **(B)**. Quantitative colocalization analysis was performed using the original-resolution image files; representative images were resized for figure presentation. Thresholds for each fluorescence channel were set consistently across all groups within each time point to minimize potential bias. Data represent analysis of three different fields per condition from one experiment. Statistical analysis was performed in GraphPad Prism using two-way ANOVA with *post-hoc* tests, and significance is indicated by asterisks (*p < 0.05). (See **Supplementary Figure 1** for corresponding BHK images.).

Rab14 is a small GTPase found in early and recycling endosome trafficking and has been linked to alternative sorting pathways. To identify whether FcγRIIA signalling influences Rab14 engagement, we examined CD32A–Rab14 colocalization in WT, CT, and BHK cells under mock, virus-only, and ADE conditions at 5- and 30-min post-infection with DENV, ZIKV, and JEV under ADE conditions using dengue IgG-positive human serum (**Figure 4**). The percentage of CD32A signal overlapping with Rab14 was quantified for the colocalization analysis.

In DENV infection, at 5min post-infection, WT cells showed mild but consistent colocalization between CD32A and Rab14 under both virus-only and ADE conditions. In contrast, CT cells exhibited lower Rab14 colocalization overall, particularly under ADE conditions. There were no significant differences in colocalization at 30 min post-infection in both WT and CT. These findings suggest that while Rab14 may be involved to some extent in DENV trafficking, its role appears limited, and signalling deficiency does not seem to redirect complexes to Rab14-positive compartments.

For ZIKV, WT cells under ADE conditions showed significantly higher colocalization at 5 min in both WT and CT, which further increased at 30 min. This indicates progressive trafficking of immune complexes through Rab14-positive compartments in the presence of ITAM signalling of FcγRIIA. The CT cells showed minimal colocalization under ADE at 5 min, but this increased by 30 min timepoint, suggesting delayed Rab14 engagement in the absence of FcγRIIA signalling. Virus-only conditions also exhibited increased Rab14 overlap at 30 min in both WT and CT, though levels remained lower than ADE, pointing to an antibody-dependent enhancement of Rab14-mediated routing.

In JEV infection, WT cells under ADE conditions showed lower colocalization at 5 min but at 30min, the signal overlap of CD32A and Rab14 saw an increased trend, indicating time-dependent recruitment of this trafficking route. Interestingly, CT cells followed a different pattern, with higher colocalization at 5 min under ADE but showed decreasing trend at 30 min. Moreover, CT virus-only samples displayed higher Rab14 overlap at 5 min compared to ADE, suggesting that JEV uptake in CT cells may engage Rab14 trafficking even without immune complexes, possibly through an alternative or compensatory mechanism.

Overall, these findings indicate that Rab14 trafficking is virus- and time-dependent, with stronger involvement observed in ZIKV and JEV compared to DENV, in which Rab14 compartmentalization was less prominent. This suggests that ZIKV and JEV are associated with Rab14+ endosomes over time, with differences notable in CT cells.

To validate the findings of CD32A and Rab14 colocalization observations using dengue IgG-positive human serum, we conducted the colocalization analysis of CD32A with Rab14 using the pan-flavivirus monoclonal antibody, mAb 4G2, with ZIKV under ADE conditions (**Supplementary Figure 2**). The observations reflected a similar trend as with dengue IgG-positive serum. Compared to 5 min, WT showed increased colocalization at 30 min. CT also followed a similar trend. These results suggest altered engagement of Rab14-positive compartments during ZIKV infection under ADE conditions in the absence of FcγRIIA signalling. The relatively limited increase in ZIKV early endosomal targeting may reflect lower cross-reactive binding efficiency of dengue-immune serum toward ZIKV antigens.

### 3.6 CD32A–Rab7 colocalization reflects virus-specific late endosomal engagement

**Figure 5:**
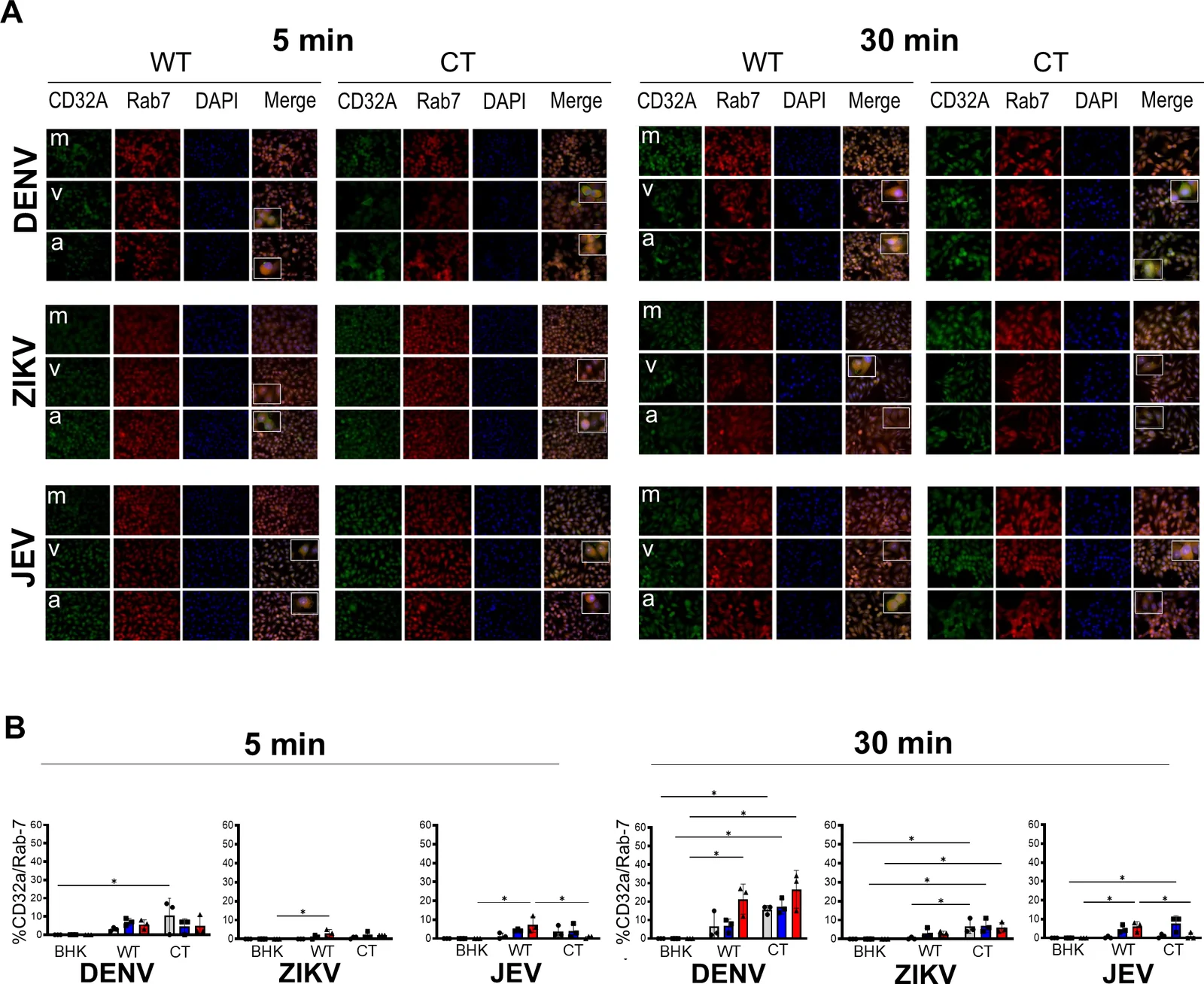
Colocalization of CD32A and Rab7 in BHK, WT, and CT cells under DENV, ZIKV, JEV infection and ADE conditions using dengue IgG-positive human serum, at 5 min- and 30 min- post-infection. Representative immunofluorescence images of wild-type FcγRIIA-expressing BHK-21 cells (WT), FcγRIIA-expressing BHK-21 cells lacking the cytoplasmic region (CT) at 5 min post-infection and 30 min post-infection under DENV, ZIKV, JEV ADE conditions **(A)**. Cells were treated under mock (m), virus-only (MOI 0.1) (v), or ADE conditions (DENV/ZIKV/JEV, MOI 0.1 with dengue IgG-positive human serum at 1:100 dilution) (a). Colocalization was analysed using Keyence BZ-X Hybrid Cell Count Software by calculating the percentage of CD32A-positive signal overlapping with the Rab7 signal **(B)**. Quantitative colocalization analysis was performed using the original-resolution image files; representative images were resized for figure presentation. Thresholds for each fluorescence channel were set consistently across all groups within each time point to minimize potential bias. Data represent analysis of three different fields per condition from one experiment. Statistical analysis was performed in GraphPad Prism using two-way ANOVA with *post-hoc* tests, and significance is indicated by asterisks (*p < 0.05). (See **Supplementary Figure 1** for corresponding BHK images.).

To determine the involvement of late endosomes in ADE-related viral trafficking, we evaluated the colocalization between CD32A and Rab7, a marker of late endosomes, at 5- and 30-mins post-infection under mock, virus-only, and ADE conditions at 5- and 30- min post-infection with DENV, ZIKV, and JEV under ADE conditions using dengue IgG-positive human serum (**Figure 5**). The percentage of CD32A colocalization with Rab7 was quantified for the colocalization analysis.

In DENV infection, Rab7 colocalization was found to be minimal at 5 min across WT, CT and BHK cells, as expected for an early timepoint. When reaching 30 minutes, WT cells under ADE conditions showed a significant increase in Rab7 colocalization, suggesting efficient progression to late endosomes via FcγRIIA-mediated signalling. CT cells also showed significantly elevated colocalization under ADE at 30 minutes, when compared to BHK ADE, but the increase was not statistically significant compared to that of WT ADE. This suggests that Rab7 trafficking still occurs in the absence of FcγRIIA signalling, though it may be delayed or less coordinated.

In ZIKV infection, the pattern was more subtle. Rab7 colocalization remained low in all groups at 5 minutes. At 30 minutes, CT cells under ADE conditions showed a significantly increased overlap, compared to BHK ADE signals, but this difference was not significant with that of WT ADE. WT cells showed only minimal Rab7 engagement under ADE at this point. These results indicate that ZIKV–antibody complexes can access late endosomes even in CT cells, but this process may be less dependent on canonical FcγRIIA signalling.

In JEV infection, colocalization was generally low across all groups and timepoints. WT cells under ADE showed a relatively higher percentage of Rab7 colocalization at both 5 min and 30 min compared to other conditions and this was significant compared to that of BHK and CT observations. CT cells remained consistently low colocalization of Rab7 throughout, suggesting limited engagement of Rab7-positive compartments in the absence of FcγRIIA signalling. Together, these findings suggest that while DENV relies more on the FcγRIIA signalling for efficient late endosomal targeting, ZIKV and JEV appear to engage Rab7 to a lesser extent and may follow alternate or less productive trafficking routes during ADE.

To further investigate the observations of the late endosomal Rab7 colocalization with CD32A using dengue IgG-positive human serum, we performed the colocalization analysis of CD32A with Rab7 using the pan-flavivirus monoclonal antibody, mAb 4G2, with ZIKV under ADE conditions (**Supplementary Figure 2**). Interestingly, while CT cells remained in a similar association with Rab7 over time, WT tend to show decrease in colocalization at 30 min. Although no statistical test was applied at the time, this shift may suggest that late endosomal targeting under 4G2-mediated ADE is less efficient in WT cells compared to the human serum-based ADE condition. The overall virus- and marker-specific colocalization patterns are summarized in **Table 1**. This suggests that the endosomal maturation involving Rab7 may be sensitive to the nature of the immune complex, and that monoclonal antibody-mediated ADE could engage different trafficking pathways. Differences between monoclonal antibody- and serum-mediated ADE likely reflect variation in antibody composition, affinity, epitope diversity, and Fc-mediated trafficking behaviour (26–29). This pathway is consistent with established requirements for DENV fusion and productive infection in late endosomal compartments (20, 38, 43). More broadly, endolysosomal compartments function as dynamic trafficking, sorting, and signalling hubs that can influence viral fate and innate immune responses (44).

**Table 1:** Summary of colocalization of CD32A with endosomal markers in FcγRIIA-expressing BHK-21 cells (WT) and FcγRIIA-expressing BHK-21 cells, without cytoplasmic region (CT), across different flavivirus infection.

Cell type	Marker	Time	DENV	ZIKV	JEV	Interpretation
WT	EEA-1	5 min	High	Low	Moderate	Rapid early endosomal targeting for DENV; less engagement for ZIKV and JEV.
		30 min	Declined	Low–Moderate	Slightly declined	Endosomal maturation proceeds in which signal declined in DENV and similar in JEV; whereas slight increase in ZIKV.
CT		5 min	Low	Low	Low	Delayed endosomal routing; DENV, ZIKV gradually increasing at 30 min.
		30 min	Increased	Increased	Unchanged	Continued accumulation in EEA-1-positive compartments in CT cells, especially for ZIKV.
WT	IRAP	5 min	Low	Low	Low	Minimal involvement of IRAP at early stage of infection with DENV, ZIKV, JEV
		30 min	Low	Moderate	Low	ZIKV showed IRAP involvement at 30 min; DENV and JEV remain low.
CT		5 min	Low	Moderate	High	IRAP engagement for ZIKV and JEV, suggesting possible alternate trafficking.
		30 min	Low	Moderate	Declined	IRAP overlap in JEV declined by 30 min, ZIKV remained moderate.
WT	Rab14	5 min	Low	Low	Mild	Rab14 minimally involved at 5 min across all infections.
		30 min	Mild	Moderate	Moderate	Modest increase in Rab14 at 30 min, especially for ZIKV and JEV.
CT		5 min	Mild	High	Moderate	Early Rab14 signal for ZIKV and JEV, supporting potential alternate trafficking.
		30 min	Moderate	Declined	Moderate	Rab14 signal declined in ZIKV, however it remained moderate for JEV.
WT	Rab7	5 min	Low	Low	Low	Rab7 involvement was limited at 5 min in all infections.
		30 min	Moderate	Low	Low	Rab7 maturation occurred in DENV at 30 min; ZIKV and JEV remain low.
CT		5 min	Moderate	Mild	Low	Rab7 engagement was moderate to mild in DENV, ZIKV, respectively; remained low in JEV.
		30 min	Low	Moderate	Moderate	Rab7 colocalization increased by 30 min, especially in ZIKV and JEV.

### 3.7 Transcriptional profiling of FcγRIIA-mediated ADE by RNA sequencing

**Figure 6:**
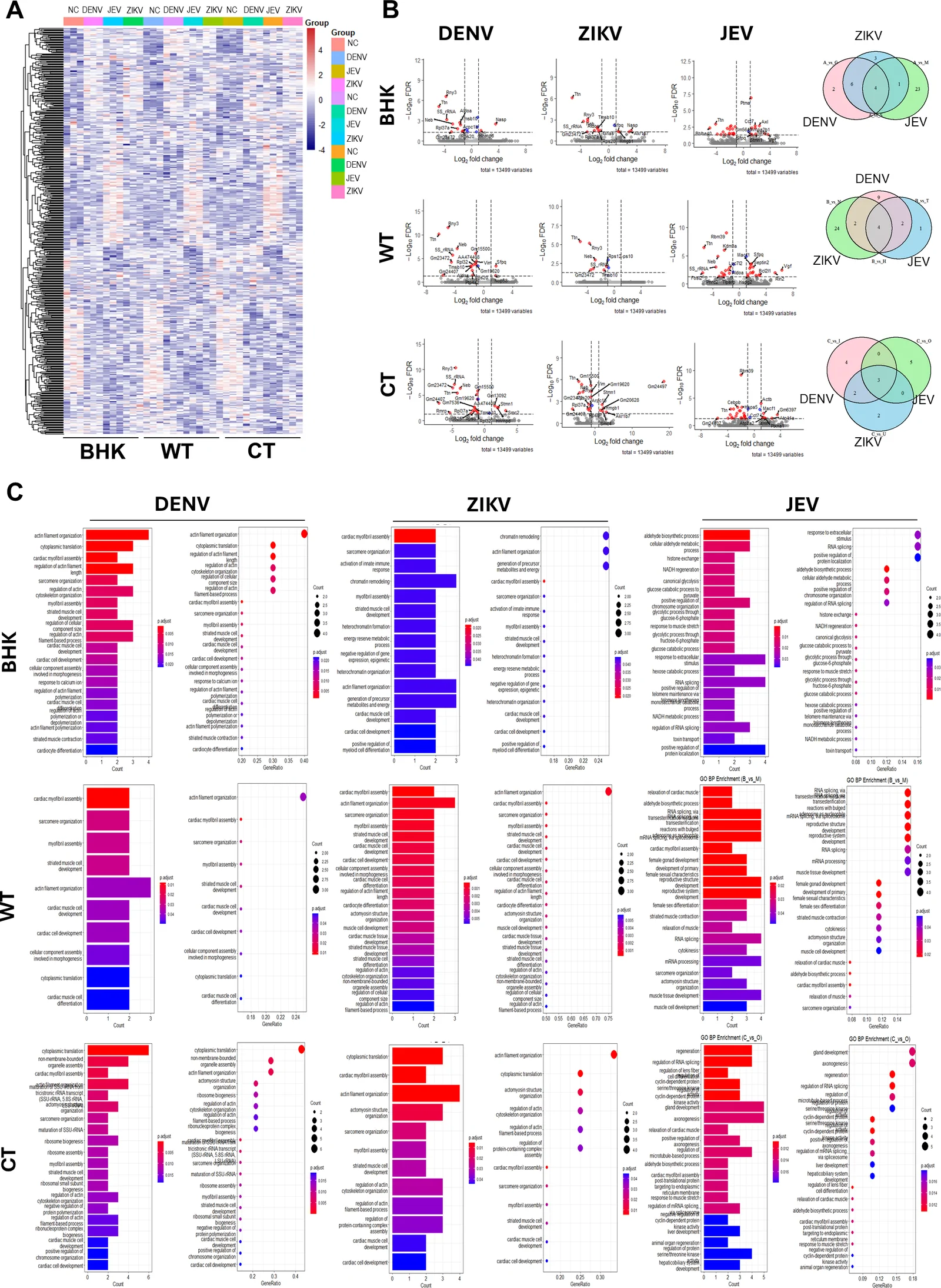
Transcriptomic profiling of FcγRIIA-expressing BHK-21 cells under ADE conditions. **(A)** Heatmap of differentially expressed genes (DEGs) across FcγRIIA-non-expressing BHK-21 (BHK), FcγRIIA-expressing BHK-21cells (WT) and FcγRIIA-expressing BHK-21 cells, without cytoplasmic domain (CT) at 48-hours post-infection (hpi) with DENV, ZIKV, and JEV compared to mock controls. Each column represents an individual sample, and clustering was performed across 13,489 genes. **(B)** Volcano plots showing log2 fold change >2, p < 0.05 in each cell type following DENV, ZIKV, JEV infection. Venn diagrams illustrate the overlap of DEGs among flaviviruses within each cell type. **(C)** Gene Ontocology (GO) enrichment analysis of DEGs in biological process categories for BHK, WT, and CT cells under DENV, ZIKV, JEV infection ADE conditions. Colour intensity reflects adjusted p-values, and circle size indicates gene counts contributing to each term. Consensus between the DEGs across flavivirus and cell types were minimal indicating that ADE in the presence of FcγRIIA, with and without the cytoplasmic region, may lead to different pathways and outcomes.

To determine the outcomes of FcγRIIA signalling on host gene expression under ADE conditions, we conducted RNA sequencing on FcγRIIA-expressing BHK-21 cells (WT), FcγRIIA-expressing BHK-21 cells without the cytoplasmic domain (CT), and FcγRIIA-non-expressing BHK-21 cells infected with DENV, ZIKV, or JEV under ADE conditions compared to mock untreated samples. RNA was collected at 24- and 48-hours post-infection (hpi) after stimulation. Principal Component Analysis (PCA) was used to examine the quality control and to identify global transcriptomic divergences between samples. The plots revealed that samples clustered primarily by virus type rather than by cell type or FcγRIIA signalling status (WT vs. CT vs. BHK), indicating that virus-specific transcriptional responses might be more dominant than receptor-mediated signalling effects at these timepoints (**Supplementary Figure 3**). Notably, WT and CT samples overlapped within the same flavivirus group, suggesting minimal transcriptional changes between them at both 24 and 48 hpi. Differential gene analysis was performed by comparing fold-changes in gene expression of FcγRIIA-expressing BHK-21 cells (WT) and FcγRIIA-expressing BHK-21 cells, without cytoplasmic domain (CT) FcγRIIA, and FcγRIIA-non-expressing BHK-21 (BHK), at 48-hours post-infection (hpi) with DENV, ZIKV, and JEV (**Figure 6**; **Supplementary Table 6**). Among the 13,489 gene set analysed, log2 fold change >2 with a p-value of <0.05 genes were as indicated (**Figure 6**). The consensus among the differentially expressed genes (DEGs) across different flaviviruses and cell types was minimal. This suggests that once a virus infection is established, the differences in gene expression by the route of entry are limited but are largely dependent on the specific virus type. These findings imply that FcγRIIA signalling may not exert a strong influence on host gene expression at later stages of infection or that the key transcriptional changes might have occurred earlier and were not captured in this analysis. Further analyses at different time points may capture FcγRIIA-associated host expression across flaviviruses.

## 4 Discussion

FcγRIIA-mediated signalling has been previously implicated in antibody-dependent enhancement (ADE) of dengue virus (DENV) infection, where the cytoplasmic domain is required for efficient signal transduction and viral replication (23). In this study, we extend these observations across multiple flaviviruses and demonstrate that FcγRIIA functions as a host-associated determinant that modulates intracellular trafficking and infection efficiency rather than acting solely as an entry receptor.

ADE was observed in both wild-type (WT) and cytoplasmic-truncated (CT) FcγRIIA-expressing cells, indicating that immune-complex binding and uptake can occur independently of intracellular signalling. However, enhancement efficiency generally was higher in WT cells and differed in a virus-dependent manner, supporting a model in which FcγRIIA signalling increases the efficiency, rather than the occurrence, of ADE. This is consistent with previous reports showing that disruption of the ITAM-containing cytoplasmic domain does not fully abrogate enhancement but reduces its efficiency (24). Together, these findings position FcγRIIA signalling as a modulatory host factor that influences infection outcomes under pre-existing immunity conditions.

**Figure 7:**
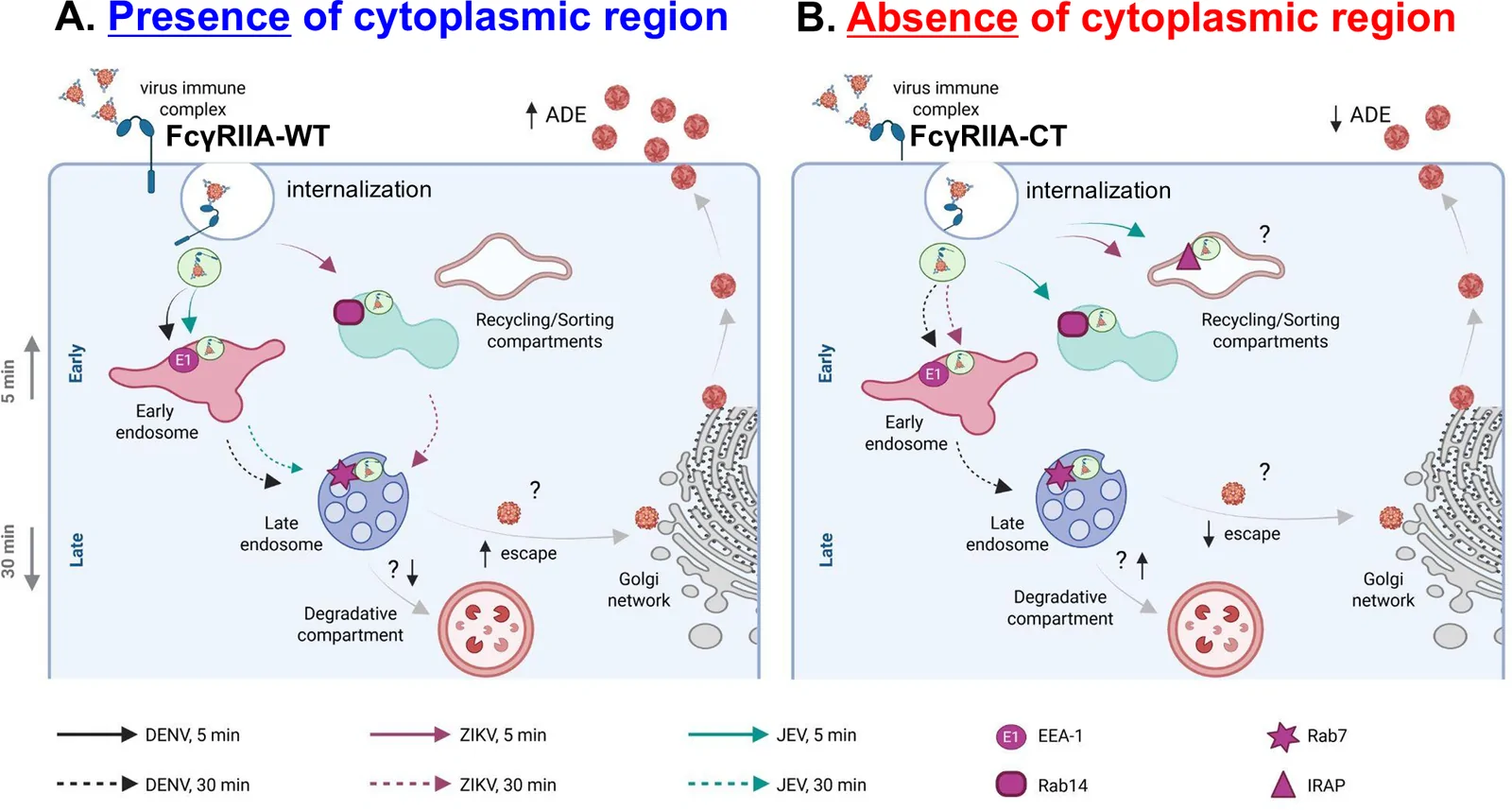
Schematic diagram of intracellular trafficking via FcγRIIA-WT and FcγRIIA-CT during flavivirus infection. **(A)** Flavivirus immune complexes (IC) were rapidly internalized and trafficked through early (EEA1+) to late (Rab7+) endosomes in FcγRIIA-expressing BHK-21 cells (FcγRIIA-WT), facilitating efficient viral fusion and potential escape to the Golgi for replication process, thereby supporting infection enhancement under ADE conditions. **(B)** In the absence of the FcγR cytoplasmic region, the ICs travelled to the endosomal compartments in a delayed manner or altered patterns. This indicates possible accumulation of the ICs in early or non-canonical compartments (e.g., IRAP or Rab14) and potentially altering the efficiency with which immune complexes reach fusion-permissive compartments. Meanwhile, in FcγRIIA-non-expressing BHK-21 cells, minimal internalization or endosomal trafficking of the ICs were observed, indicating that FcγRIIA is essential for ADE-mediated uptake in this model. These differences highlight the critical role of FcγRIIA signalling in mediating endosomal progression and differential ADE outcomes.

A key mechanistic insight from this study is that FcγRIIA signalling regulates the kinetics and routing of virus–antibody complexes through the endosomal network. WT cells exhibited rapid trafficking into early endosomes (EEA1^+^) within minutes of infection, followed by progression to Rab7^+^ late endosomes, particularly for DENV. A schematic summary of the proposed FcγRIIA-dependent trafficking model is shown in **Figure 7**. This pathway is consistent with established requirements for DENV fusion and productive infection (20, 38, 43). In contrast, CT cells displayed delayed early endosomal engagement and reduced coordination of endosomal maturation, similar to observations in other FcγR-dependent viral systems such as Ebola virus (35). These findings indicate that FcγRIIA signalling enhances the efficiency of canonical endocytic trafficking required for viral fusion.

Importantly, virus-specific differences were observed, highlighting the interaction between host-associated factors and intrinsic viral entry programs. DENV showed strong dependence on FcγRIIA signalling for efficient early-to-late endosomal progression, whereas ZIKV and JEV exhibited more flexible trafficking patterns. ZIKV demonstrated delayed and less efficient early endosomal targeting, consistent with reports of alternative entry pathways and virus-induced cellular remodelling (45–47, 51, 59). JEV displayed substantial early endosomal localization even in the absence of ADE, reflecting its ability to utilize multiple entry routes depending on cellular context (49, 50). These virus-specific differences should also be interpreted in the broader context of FcγR biology, as Fcγ receptors regulate antibody effector functions, immune-cell activation, and downstream signalling outcomes beyond simple ligand binding (48, 54). These differences suggest that the impact of FcγRIIA signalling on ADE is virus-dependent and shaped by inherent viral entry strategies.

Beyond canonical pathways, altered trafficking in CT cells revealed increased engagement of non-classical compartments, including IRAP^+^ and Rab14^+^ endosomes. IRAP-associated compartments have been described as specialized recycling, immune-processing, and signalling platforms for Fcγ receptors (52, 55–57). The enrichment of ZIKV immune complexes in IRAP-positive compartments in WT cells suggests that FcγRIIA signalling may promote access to these signalling-competent vesicles. In contrast, CT cells showed altered or compensatory routing into IRAP or Rab14 pathways, indicating that in the absence of signalling, immune complexes may be redirected into alternative trafficking routes. Rab14, which regulates endosome–Golgi and recycling pathways, has been implicated in non-canonical trafficking and may act as a compensatory pathway during impaired FcγRIIA signalling (53, 58).

Although murine monoclonal antibodies were initially used in this study, human dengue-immune serum demonstrated similar ADE trends, indicating that the observed FcγRIIA-associated mechanisms were reproducible across different antibody sources. Furthermore, hFcγRIIA-transfected cells consistently detected ADE activity with both murine monoclonal antibodies and human serum, suggesting that FcγRIIA engagement contributes to the early stages of infection enhancement (22, 25). Nevertheless, differences in Fcγ affinity between murine and human IgG subclasses may influence receptor binding efficiency and subsequent internalization pathways and should therefore be considered when extrapolating these findings to human biology. Further studies using human-derived or humanized monoclonal antibodies would help clarify these effects. The FcγRIIA-BHK plaque-based assay used here directly measures infectious enhancement as plaque formation following antibody-mediated infection. Although quantification of released virus in culture supernatants would provide complementary information, previous studies using this FcγR-based ADE system have shown that plaque-based enhancement activity is associated with increased virus titres in culture supernatants (61). Collectively, these findings support a model in which FcγRIIA signalling coordinates efficient intracellular routing of virus–antibody complexes, facilitating progression through endosomal compartments required for productive infection. In its absence, delayed or altered trafficking reduces the efficiency of ADE without completely preventing viral entry. This distinction is critical in the context of host-associated factors, as it suggests that variability in FcγR signalling capacity—driven by host genetics, immune history, or receptor expression—may influence susceptibility to enhanced disease.

Although the colocalization analyses identify FcγRIIA-associated trafficking patterns, they do not by themselves establish a functional hierarchy of endosomal compartments or prove that specific compartments enhance or restrict productive infection. Endolysosomal compartments are dynamic signalling and sorting hubs rather than passive degradative structures, and their membrane identity can influence viral fate, innate immune signalling, and receptor-associated trafficking outcomes (44). Therefore, the EEA1-, IRAP-, Rab14-, and Rab7-associated patterns observed here should be interpreted as trafficking-associated signatures of FcγRIIA signalling rather than direct functional evidence that these compartments determine productive infection. Beyond canonical pathways, altered trafficking in CT cells revealed increased engagement of non-classical compartments, including IRAP^+^ and Rab14^+^ endosomes. IRAP-associated compartments have been described as specialized recycling, immune-processing, and signalling platforms for Fcγ receptors (52, 55–57). However, the present colocalization data do not establish whether IRAP-positive compartments enhance, restrict, or merely accompany immune-complex trafficking during ADE. Similarly, Rab14 regulates multiple trafficking programs, including endosome–Golgi transport, recycling, and endosomal maturation (53, 58). Therefore, Rab14 association in CT cells should be interpreted as altered compartment engagement rather than definitive evidence of compensatory trafficking. Further functional studies will be required to determine whether these compartments directly influence productive infection.

Consistent with this interpretation, transcriptomic analysis revealed minimal differences between WT and CT cells at later time points, indicating that FcγRIIA-mediated effects are primarily exerted during early post-entry stages. Previous studies have shown that ADE can alter downstream immune responses, cytokine profiles, and transcriptional programs (41, 42, 60). Our data suggest that the dominant contribution of FcγRIIA signalling lies in regulating trafficking dynamics rather than sustained transcriptional reprogramming at later stages. Together, these findings support a model in which FcγRIIA signalling modulates ADE magnitude and trafficking kinetics in a virus-dependent manner rather than acting as an absolute requirement for viral uptake. Other host-associated factors, including FcγR expression level, FcγR genetic variation, IgG subclass distribution, immune history, and downstream signalling capacity, may further modulate FcγRIIA-dependent ADE. These factors may influence receptor engagement, immune-complex uptake, and endosomal routing efficiency.

Overall, this study identifies FcγRIIA signalling as a host-associated factor that links immune complex recognition to intracellular trafficking efficiency during flavivirus infection. By shaping the kinetics and routing of viral entry, FcγRIIA contributes to differential ADE outcomes across viruses. These findings have direct implications for vaccine and antibody-based strategies, where pre-existing immunity and Fc receptor engagement may influence both protective and pathogenic responses.

## Data Availability

The datasets presented in this study can be found in online repositories. The names of the repository/repositories and accession number(s) can be found below: https://zenodo.org/uploads/17415336, 10.5281/zenodo.17415336.

## Supplementary material

Supplementary Figure 1 Colocalization of CD32A and EEA-1 **(A)**, IRAP **(B)**, Rab14 **(C)**, and Rab7 **(D)** in BHK cells under DENV, ZIKV, JEV infection and ADE conditions using dengue IgG-positive human serum, at 5 min- and 30 min- post-infection. Representative immunofluorescence images of FcγRIIA-non-expressing BHK-21 cells (BHK) at 5 min post-infection and 30 min post-infection under DENV, ZIKV, JEV ADE conditions. Cells were treated under mock (m), virus-only (MOI 0.1) (v), or ADE conditions (DENV/ZIKV/JEV, MOI 0.1 with dengue IgG-positive human serum at 1:100 dilution) (a). Colocalization was analysed using Keyence BZ-X Hybrid Cell Count Software by calculating the percentage of CD32A-positive signal overlapping with the EEA-1, IRAP, Rab14, Rab7 signal. Thresholds for each fluorescence channel were set consistently across all groups within each time point to minimize potential bias.

Supplementary Figure 2 Colocalization of CD32A and EEA-1, IRAP, Rab14, Rab7 in BHK, WT, and CT cells under ADE conditions using ZIKV and mAb 4G2, at 5 min- and 30 min- post-infection. **(A)** Representative immunofluorescence images of wild-type FcγRIIA-expressing BHK-21 cells (WT), FcγRIIA-expressing BHK-21 cells lacking the cytoplasmic region (CT) at 5 min- and 30 min- post-infection. Cells were treated under ADE conditions (ZIKV, MOI 0.1 with monoclonal antibody 4G2 at 1:100 dilution). **(B)** Quantification of colocalization at 5 min- and 30min- post-infection in BHK (black), WT (blue), CT (red). Colocalization was analysed using Keyence BZ-X Hybrid Cell Count Software by calculating the percentage of CD32A-positive signal overlapping with the EEA-1, IRAP, Rab14 and Rab7 signals. Thresholds for each fluorescence channel were set consistently across all groups within each time point to minimize bias. Three replicates were analysed for each experiment. Statistical analysis was performed in GraphPad Prism.

Supplementary Figure 3 Differential expressed gene (DEG) across flavivirus infection at 48-hours post infection. **(A)** Differentially expressed genes (DEGs) in FcγRIIA-non-expressing BHK-21 cells (BHK), FcγRIIA-expressing BHK-21 cells (WT) and FcγRIIA-expressing BHK-21 cells, without cytoplasmic domain (CT) at 48 hours post-infection (hpi) with DENV-, ZIKV- and JEV- ADE conditions, representative of 3 replicates of each assay. Among the 13,489 gene set analysed, log2 fold change >2 with a p-value of <0.05 genes were counted of both up-regulated and down-regulated genes. Each dot represents an individual DEG. **(B)** Principal Component Analysis (PCA) was performed on the RNAseq data of the FcγRIIA-expressing BHK-21cells (WT) and FcγRIIA-expressing BHK-21 cells, without cytoplasmic domain (CT), and FcγRIIA-non-expressing BHK-21 (BHK), at 24- and 48-hours post-infection (hpi) with DENV, ZIKV, and JEV. The plot visualizes the variance in gene expression profiles among the cell types and viruses. Clustering patterns indicate the degree of similarity in transcriptomic responses across different experimental conditions. The PCA showed minimal transcriptional differences between WT and CT cells, with some degree of differential genes expression observed across flaviviral infections and with the progress of infection from 24- to 48-hours.

Supplementary Table 1 Primers used in PCR amplification of pcDNA3.1/neo+ vector insert.

Supplementary Table 2 Summary of endosomal markers used in this study.

Supplementary Table 3 Absolute plaque counts underlying DENV 12D11 ADE fold-enhancement assay. Absolute plaque counts are shown as mean plaques per well under each antibody or serum dilution. Fold enhancement was calculated by dividing the mean plaque number in the presence of antibody or serum by the mean plaque number in the no-antibody control for the corresponding cell type. These values represent plaque-based infectious output in the ADE assay and are not culture-supernatant virus titres or back-calculated viral stock titres. BHK cells are FcγRIIA-negative non-Fc controls.

Supplementary Table 4 Absolute plaque counts underlying DENV 4G2 ADE fold-enhancement assay. Absolute plaque counts are shown as mean plaques per well under each antibody or serum dilution. Fold enhancement was calculated by dividing the mean plaque number in the presence of antibody or serum by the mean plaque number in the no-antibody control for the corresponding cell type. These values represent plaque-based infectious output in the ADE assay and are not culture-supernatant virus titres or back-calculated viral stock titres. BHK cells are FcγRIIA-negative non-Fc controls.

Supplementary Table 5 Absolute plaque counts underlying DENV human dengue IgG-positive serum ADE fold-enhancement assay. Absolute plaque counts are shown as mean plaques per well under each antibody or serum dilution. Fold enhancement was calculated by dividing the mean plaque number in the presence of antibody or serum by the mean plaque number in the no-antibody control for the corresponding cell type. These values represent plaque-based infectious output in the ADE assay and are not culture-supernatant virus titres or back-calculated viral stock titres. BHK cells are FcγRIIA-negative non-Fc controls.

Supplementary Table 6 Number of differentially expressed genes (DEGs) in FcγRIIA-non-expressing BHK-21 cells (BHK), FcγRIIA-expressing BHK-21 cells (WT) and FcγRIIA-expressing BHK-21 cells, without cytoplasmic domain (CT), at 48-hours post-infection (hpi) with DENV, ZIKV, and JEV. Among the 13,489 gene setanalysed, log2 fold change >2 with a p-value of <0.05 genes were determined in comparison to the non-infected cell lines. *DEGs = Differentially expressed genes
