# Correction: FcγRIIA signalling as a host-associated determinant of endosomal trafficking and antibody-dependent enhancement in flavivirus infection

**DOI:** 10.3389/fimmu.2026.1926257

**Published:** 2026-08-21

**Authors:** Nensar Wai Wai Phyo Rakwi, Daisuke Muraoka, Kazumi Haga, Adeline Syin Lian Yeo, Meng Ling Moi

**Affiliations:** 1 Department of Developmental Medical Sciences, School of International Health, Graduate School of Medicine, The University of Tokyo, Tokyo, Japan; 2 Division of Immune Response, Aichi Cancer Center Research Institute, Nagoya, Japan

**Keywords:** antibody-dependent enhancement (ADE), endosomal trafficking, FcγRIIa (CD32A), flavivirus (DENV ZIKV JEV), host-associated factors, immune complexes, pre-existing immunity, viral pathogenesis

The **Ethics statement** was erroneously given as “This study was approved by the Institutional Review Board of the Graduate School of Medicine, the University of Tokyo (2023313NI, 2025168NI). The studies were conducted in accordance with the local legislation and institutional requirements. The human samples used in this study were acquired from primarily isolated as part of your previous study for which ethical approval was obtained. Written informed consent for participation was not required from the participants or the participants’ legal guardians/next of kin in accordance with the national legislation and institutional requirements. Ethical approval was not required for the studies on animals in accordance with the local legislation and institutional requirements because only commercially available established cell lines were used.”

The correct **Ethics statement** is “This study was approved by the Institutional Review Board of the Graduate School of Medicine, The University of Tokyo (2023313NI, 2025168NI). The study was conducted in accordance with local legislation and institutional requirements. Human-derived samples used in this study were obtained under the above ethical approvals. Written informed consent was not required in accordance with national legislation and institutional requirements. No animal experiments were conducted in this study. Experiments were performed using established cell lines; therefore, additional animal ethics approval was not required.”

The original version of this article has been updated.

