# Supplementary figures and images for: FcγRIIA signalling as a host-associated determinant of endosomal trafficking and antibody-dependent enhancement in flavivirus infection
Source: Front Immunol. 2026 Jun 17;17:1854518. doi: 10.3389/fimmu.2026.1854518 (PMC13318605; doi:10.3389/fimmu.2026.1854518)

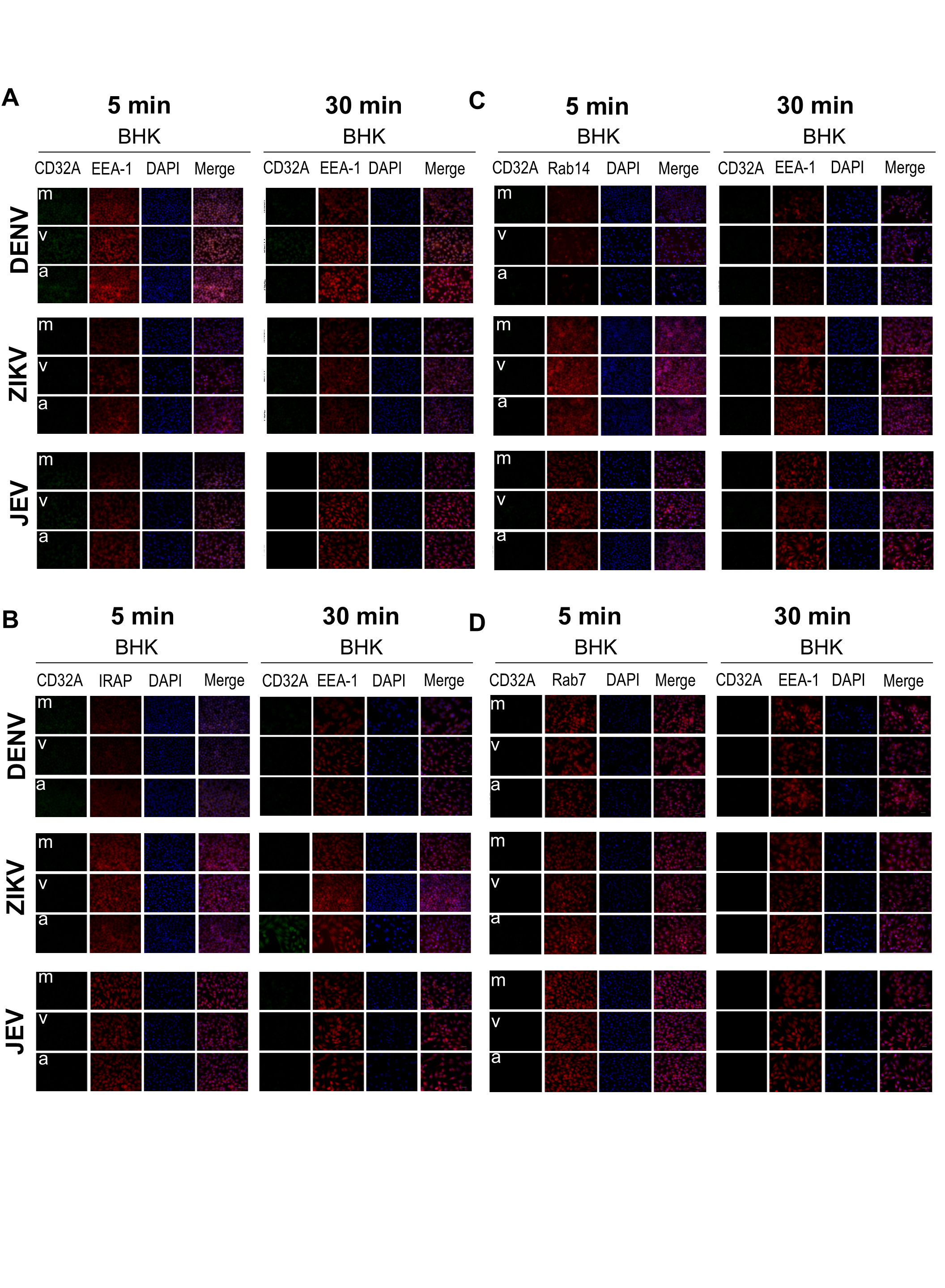

Supplement: Supplementary Figure 1 — Colocalization of CD32A and EEA-1 (A), IRAP (B), Rab14 (C), and Rab7 (D) in BHK cells under DENV, ZIKV, JEV infection and ADE conditions using dengue IgG-positive human serum, at 5 min- and 30 min- post-infection. Representative immunofluorescence images of FcγRIIA-non-expressing BHK-21 cells (BHK) at 5 min post-infection and 30 min post-infection under DENV, ZIKV, JEV ADE conditions. Cells were treated under mock (m), virus-only (MOI 0.1) (v), or ADE conditions (DENV/ZIKV/JEV, MOI 0.1 with dengue IgG-positive human serum at 1:100 dilution) (a). Colocalization was analysed using Keyence BZ-X Hybrid Cell Count Software by calculating the percentage of CD32A-positive signal overlapping with the EEA-1, IRAP, Rab14, Rab7 signal. Thresholds for each fluorescence channel were set consistently across all groups within each time point to minimize potential bias. [file Image1.png]

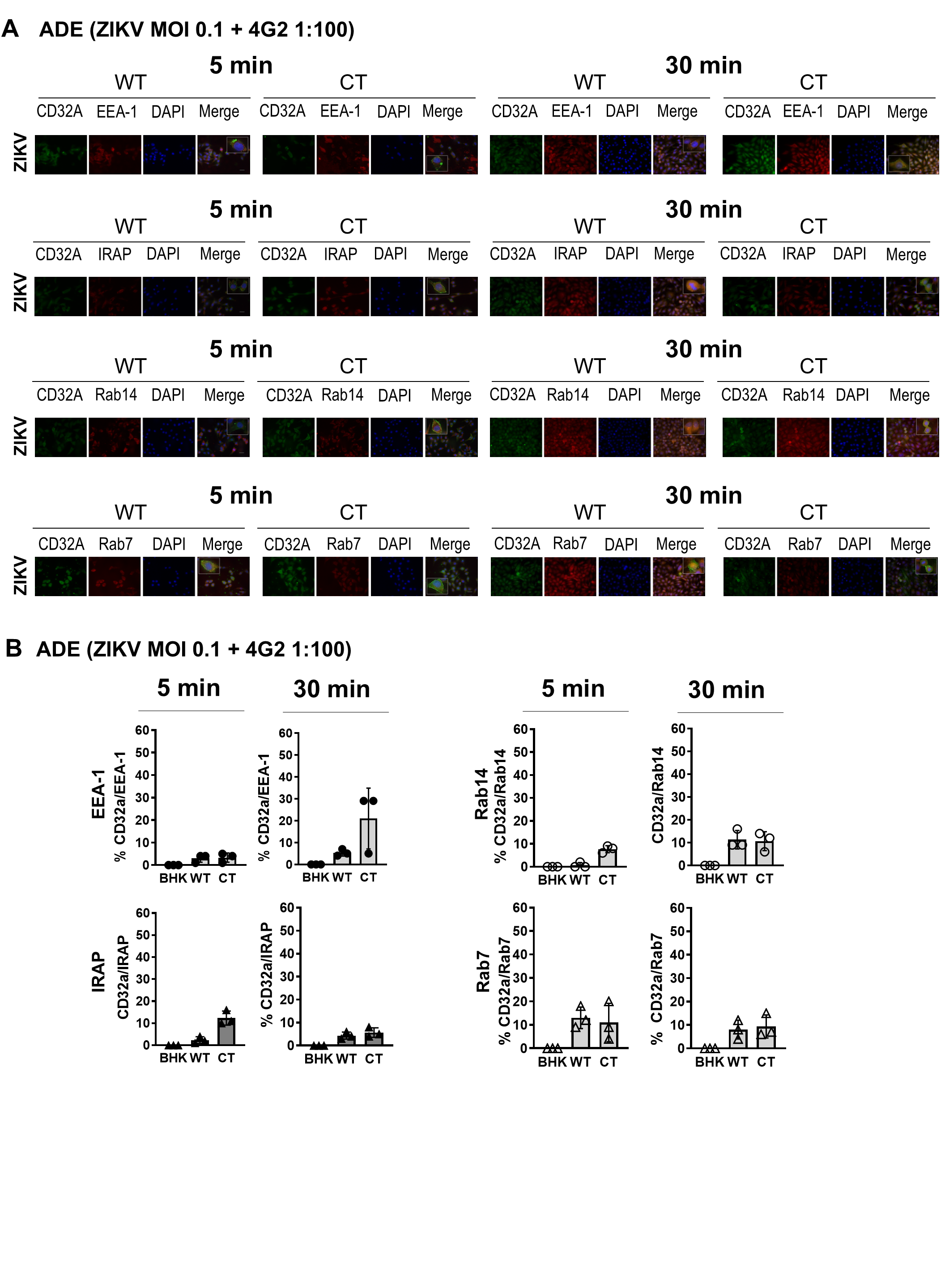

Supplement: Supplementary Figure 2 — Colocalization of CD32A and EEA-1, IRAP, Rab14, Rab7 in BHK, WT, and CT cells under ADE conditions using ZIKV and mAb 4G2, at 5 min- and 30 min- post-infection. (A) Representative immunofluorescence images of wild-type FcγRIIA-expressing BHK-21 cells (WT), FcγRIIA-expressing BHK-21 cells lacking the cytoplasmic region (CT) at 5 min- and 30 min- post-infection. Cells were treated under ADE conditions (ZIKV, MOI 0.1 with monoclonal antibody 4G2 at 1:100 dilution). (B) Quantification of colocalization at 5 min- and 30min- post-infection in BHK (black), WT (blue), CT (red). Colocalization was analysed using Keyence BZ-X Hybrid Cell Count Software by calculating the percentage of CD32A-positive signal overlapping with the EEA-1, IRAP, Rab14 and Rab7 signals. Thresholds for each fluorescence channel were set consistently across all groups within each time point to minimize bias. Three replicates were analysed for each experiment. Statistical analysis was performed in GraphPad Prism. [file Image2.png]

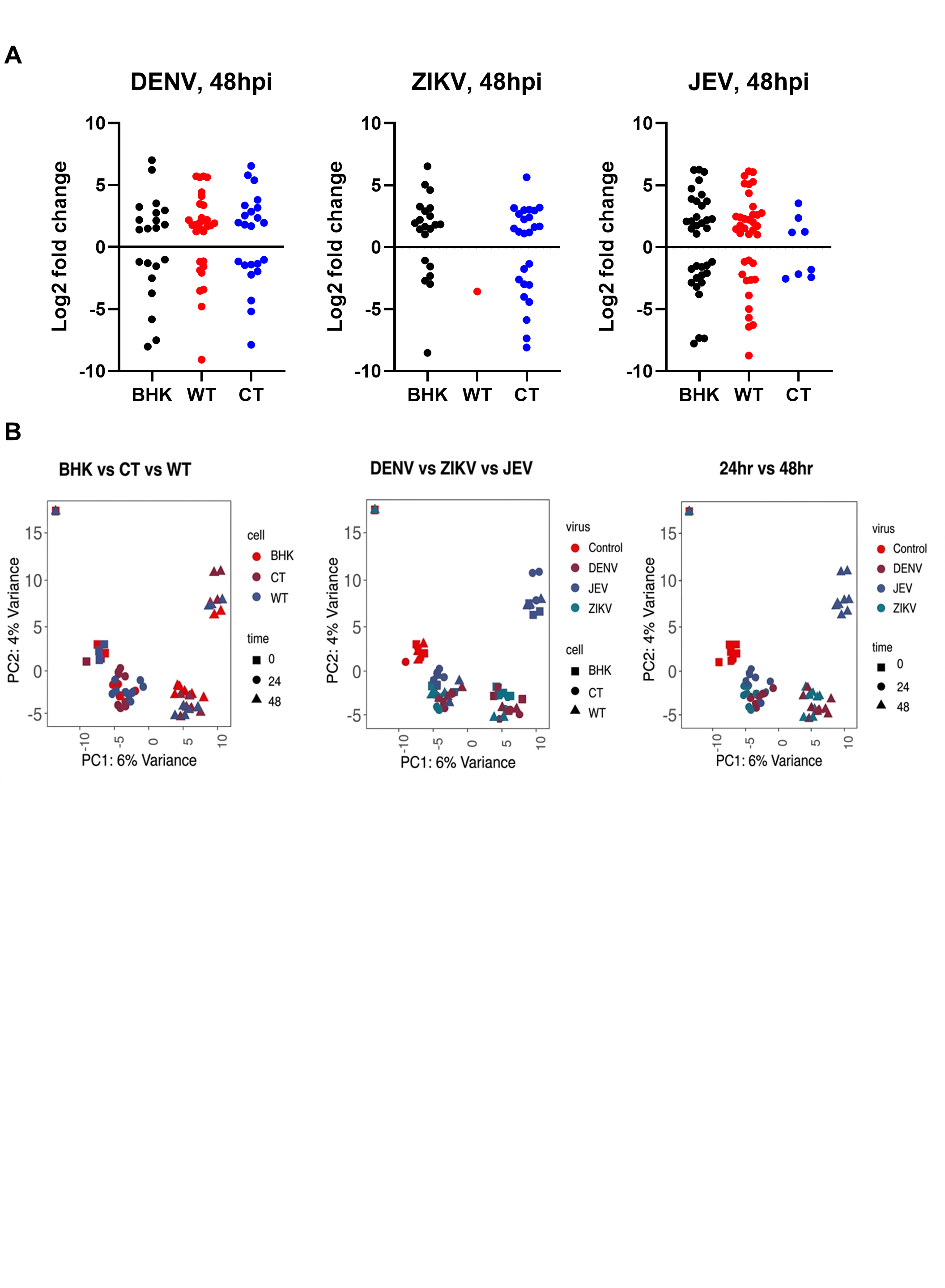

Supplement: Supplementary Figure 3 — Differential expressed gene (DEG) across flavivirus infection at 48-hours post infection. (A) Differentially expressed genes (DEGs) in FcγRIIA-non-expressing BHK-21 cells (BHK), FcγRIIA-expressing BHK-21 cells (WT) and FcγRIIA-expressing BHK-21 cells, without cytoplasmic domain (CT) at 48 hours post-infection (hpi) with DENV-, ZIKV- and JEV- ADE conditions, representative of 3 replicates of each assay. Among the 13,489 gene set analysed, log2 fold change >2 with a p-value of <0.05 genes were counted of both up-regulated and down-regulated genes. Each dot represents an individual DEG. (B) Principal Component Analysis (PCA) was performed on the RNAseq data of the FcγRIIA-expressing BHK-21cells (WT) and FcγRIIA-expressing BHK-21 cells, without cytoplasmic domain (CT), and FcγRIIA-non-expressing BHK-21 (BHK), at 24- and 48-hours post-infection (hpi) with DENV, ZIKV, and JEV. The plot visualizes the variance in gene expression profiles among the cell types and viruses. Clustering patterns indicate the degree of similarity in transcriptomic responses across different experimental conditions. The PCA showed minimal transcriptional differences between WT and CT cells, with some degree of differential genes expression observed across flaviviral infections and with the progress of infection from 24- to 48-hours. [file Image3.png]
