# Supplementary material for: FcγRIIA signalling as a host-associated determinant of endosomal trafficking and antibody-dependent enhancement in flavivirus infection
Source: Front Immunol. 2026 Jun 17;17:1854518. doi: 10.3389/fimmu.2026.1854518 (PMC13318605; doi:10.3389/fimmu.2026.1854518)
Supplement: Supplementary Table 1 — Primers used in PCR amplification of pcDNA3.1/neo+ vector insert. [file SupplementaryFile1.docx]

**Supplementary Table 1.** Primers used in PCR amplification of pcDNA3.1/neo+ vector insert

| Target | Sequence |
| --- | --- |
| T7 promoter | 5´- TAATACGACTCACTATAGGG- 3’ |
| BGH gene | 5' - TAGAAGGCACAGTCGAGG - 3' |

**Supplementary Table 2.** Summary of endosomal markers used in this study

| Marker | Full Name | Intracellular localization | Functional Role | Relevance to flavivirus infection/ADE |
| --- | --- | --- | --- | --- |
| EEA-1 | Early endosome antigen 1 | Early endosomes | Serves as a tethering protein that helps endocytic vesicles dock and fuse; central to sorting incoming cargo | Entry point for virus-antibody complexes; rapid recruitment supports early endocytic uptake via FcγRs |
| IRAP | Insulin-regulated aminopeptidase | Specialized early/recycling endosomes | Helps regulate the recycling route and slows endosomal maturation; also involved in immune processing | May prolong early endosomal residency of virus-antibody complexes, facilitating ADE in immune cells |
| Rab14 | Ras-related protein Rab-14 | Early endosomes, trans-Golgi network | Coordinates endosome-to-Golgi and recycling trafficking; maintains early endosomal compartments | Rab14 supports viral assembly; may also shape endosomal environment for flavivirus entry |
| Rab7 | Ras-related protein Rab-7a | Late endosome; transiently recruited to early endosomes | Key player in the transition from early to late endosomes; regulates degradation and endosome-lysosome fusion | Flaviviruses like DENV rely on Rab7-positive compartments for genome release; ADE, enhances targeting to late endosomes |

**Supplementary Table 3.** Absolute plaque counts underlying DENV 12D11 ADE fold-enhancement assay

| **Cell type** | **FcγRIIA status** | **Dilution** | **Mean plaques/well** | **Fold enhancement** |
| --- | --- | --- | --- | --- |
| BHK | Non-Fc control | 1:10 | 5.50 | 1.37 |
|  |  | 1:10² | 5.75 | 1.44 |
|  |  | 1:10³ | 5.00 | 1.25 |
|  |  | 1:10⁴ | 7.50 | 1.88 |
|  |  | 1:10⁵ | 7.00 | 1.75 |
|  |  | No antibody | 4.00 | 1.00 |
| WT | FcγRIIA-WT | 1:10 | 11.00 | 0.77 |
|  |  | 1:10² | 14.00 | 0.98 |
|  |  | 1:10³ | 16.25 | 1.14 |
|  |  | 1:10⁴ | 30.00 | 2.11 |
|  |  | 1:10⁵ | 22.00 | 1.54 |
|  |  | No antibody | 14.25 | 1.00 |
| CT | FcγRIIA-CT | 1:10 | 7.00 | 0.53 |
|  |  | 1:10² | 8.17 | 0.61 |
|  |  | 1:10³ | 17.17 | 1.29 |
|  |  | 1:10⁴ | 18.33 | 1.38 |
|  |  | 1:10⁵ | 14.50 | 1.09 |
|  |  | No antibody | 13.33 | 1.00 |

Absolute plaque counts are shown as mean plaques per well under each antibody or serum dilution. Fold enhancement was calculated by dividing the mean plaque number in the presence of antibody or serum by the mean plaque number in the no-antibody control for the corresponding cell type. These values represent plaque-based infectious output in the ADE assay and are not culture-supernatant virus titers or back-calculated viral stock titers. BHK cells are FcγRIIA-negative non-Fc controls.

**Supplementary Table 4.** Absolute plaque counts underlying DENV 4G2 ADE fold-enhancement assay

| **Cell type** | **FcγRIIA status** | **Dilution** | **Mean plaques/well** | **Fold enhancement** |
| --- | --- | --- | --- | --- |
| BHK | Non-Fc control | 1:10 | 7.00 | 0.58 |
|  |  | 1:10² | 8.50 | 0.71 |
|  |  | 1:10³ | 7.50 | 0.63 |
|  |  | 1:10⁴ | 8.50 | 0.71 |
|  |  | 1:10⁵ | 11.50 | 0.96 |
|  |  | No antibody | 12.00 | 1.00 |
| WT | FcγRIIA-WT | 1:10 | 2.00 | 0.27 |
|  |  | 1:10² | 10.00 | 1.33 |
|  |  | 1:10³ | 13.00 | 1.73 |
|  |  | 1:10⁴ | 11.00 | 1.47 |
|  |  | 1:10⁵ | 7.00 | 0.93 |
|  |  | No antibody | 7.50 | 1.00 |
| CT | FcγRIIA-CT | 1:10 | 5.00 | 0.63 |
|  |  | 1:10² | 18.50 | 2.31 |
|  |  | 1:10³ | 13.50 | 1.69 |
|  |  | 1:10⁴ | 8.50 | 1.06 |
|  |  | 1:10⁵ | 9.00 | 1.13 |
|  |  | No antibody | 8.00 | 1.00 |

Absolute plaque counts are shown as mean plaques per well under each antibody or serum dilution. Fold enhancement was calculated by dividing the mean plaque number in the presence of antibody or serum by the mean plaque number in the no-antibody control for the corresponding cell type. These values represent plaque-based infectious output in the ADE assay and are not culture-supernatant virus titers or back-calculated viral stock titers. BHK cells are FcγRIIA-negative non-Fc controls.

**Supplementary Table 5.** Absolute plaque counts underlying DENV human dengue IgG-positive serum ADE fold-enhancement assay

| **Cell type** | **FcγRIIA status** | **Dilution** | **Mean plaques/well** | **Fold enhancement** |
| --- | --- | --- | --- | --- |
| BHK | Non-Fc control | 1:10 | 0.75 | 0.27 |
|  |  | 1:10² | 6.75 | 2.46 |
|  |  | 1:10³ | 6.75 | 2.46 |
|  |  | 1:10⁴ | 6.75 | 2.46 |
|  |  | 1:10⁵ | 5.25 | 1.91 |
|  |  | No antibody | 2.75 | 1.00 |
| WT | FcγRIIA-WT | 1:10 | 0.25 | 0.03 |
|  |  | 1:10² | 52.25 | 6.97 |
|  |  | 1:10³ | 35.75 | 4.77 |
|  |  | 1:10⁴ | 20.25 | 2.70 |
|  |  | 1:10⁵ | 12.00 | 1.60 |
|  |  | No antibody | 7.50 | 1.00 |
| CT | FcγRIIA-CT | 1:10 | 1.00 | 0.11 |
|  |  | 1:10² | 49.75 | 5.38 |
|  |  | 1:10³ | 52.50 | 5.68 |
|  |  | 1:10⁴ | 20.25 | 2.19 |
|  |  | 1:10⁵ | 14.75 | 1.60 |
|  |  | No antibody | 9.25 | 1.00 |

Absolute plaque counts are shown as mean plaques per well under each antibody or serum dilution. Fold enhancement was calculated by dividing the mean plaque number in the presence of antibody or serum by the mean plaque number in the no-antibody control for the corresponding cell type. These values represent plaque-based infectious output in the ADE assay and are not culture-supernatant virus titers or back-calculated viral stock titers. BHK cells are FcγRIIA-negative non-Fc controls.

**Supplementary Table 6.** Number of differentially expressed genes (DEGs) in FcγRIIA-non-expressing BHK-21 cells (BHK), FcγRIIA-expressing BHK-21 cells (WT) and FcγRIIA-expressing BHK-21 cells, without cytoplasmic domain (CT), at 48-hours post-infection (hpi) with DENV, ZIKV, and JEV. Among the 13,489 gene set analysed, log2 fold change >2 with a p-value of <0.05 genes were determined in comparison to the non-infected cell lines.

| **Virus** | **Cell type** | **Number  of DEGs*** |
| --- | --- | --- |
| **DENV** | BHK | 30 |
|  | WT | 39 |
|  | CT | 30 |
| **JEV** | BHK | 42 |
|  | WT | 42 |
|  | CT | 8 |
| **ZIKV** | BHK | 26 |
|  | WT | 1 |
|  | CT | 31 |
|  |  |  |

*DEGs = Differentially expressed genes
